# Exploiting Lactoferricin (17–30) as a Potential Antimicrobial and Antibiofilm Candidate Against Multi-Drug-Resistant Enteroaggregative *Escherichia coli*

**DOI:** 10.3389/fmicb.2020.575917

**Published:** 2020-09-18

**Authors:** Jess Vergis, Satyaveer Singh Malik, Richa Pathak, Manesh Kumar, Sunitha Ramanjaneya, Nitin Vasantrao Kurkure, Sukhadeo Baliram Barbuddhe, Deepak Bhiwa Rawool

**Affiliations:** ^1^Division of Veterinary Public Health, ICAR-Indian Veterinary Research Institute, Izatnagar, India; ^2^Department of Veterinary Pathology, Nagpur Veterinary College, Nagpur, India; ^3^ICAR-National Research Centre on Meat, Hyderabad, India

**Keywords:** antimicrobial peptide, biofilm, confocal microscopy, enteroaggregative *E. coli*, *Galleria mellonella*, lactoferricin (17–30)

## Abstract

The study evaluated the *in vitro* antimicrobial and antibiofilm efficacy of an antimicrobial peptide (AMP), lactoferricin (17–30) [Lfcin (17–30)], against biofilm-forming multi-drug-resistant (MDR) strains of enteroaggregative *Escherichia coli* (EAEC), and subsequently, the *in vivo* antimicrobial efficacy was assessed in a *Galleria mellonella* larval model. Initially, minimum inhibitory concentration (MIC; 32 μM), minimum bactericidal concentration (MBC; 32 μM), and minimum biofilm eradication concentration (MBEC; 32 μM) of Lfcin (17–30) were determined against MDR-EAEC field isolates (*n* = 3). Lfcin (17–30) was tested stable against high-end temperatures (70 and 90°C), physiological concentration of cationic salts (150 mM NaCl and 2 mM MgCl_2_), and proteases (proteinase-K and lysozyme). Further, at lower MIC, Lfcin (17–30) proved to be safe for sheep RBCs, secondary cell lines (HEp-2 and RAW 264.7), and beneficial gut lactobacilli. In the *in vitro* time-kill assay, Lfcin (17–30) inhibited the MDR-EAEC strains 3 h post-incubation, and the antibacterial effect was due to membrane permeation of Lfcin (17–30) in the inner and outer membranes of MDR-EAEC. Furthermore, in the *in vivo* experiments, *G. mellonella* larvae treated with Lfcin (17–30) exhibited an increased survival rate, lower MDR-EAEC counts (*P* < 0.001), mild to moderate histopathological changes, and enhanced immunomodulatory effect and were safe to larval cells when compared with infection control. Besides, Lfcin (17–30) proved to be an effective antibiofilm agent, as it inhibited and eradicated the preformed biofilm formed by MDR-EAEC strains in a significant (*P* < 0.05) manner both by microtiter plate assay and live/dead bacterial quantification-based confocal microscopy. We recommend further investigation of Lfcin (17–30) in an appropriate animal model before its application in target host against MDR-EAEC strains.

## Introduction

In recent times, enteroaggregative *Escherichia coli* (EAEC) has been regarded as an emerging foodborne pathogen, and it has been frequently associated with the epidemic as well as endemic diarrheal episodes (Lima et al., 2018). In human infants, EAEC damages the intestinal epithelium, leading to poor nutritional status and thereby intellectual deficits (Lima et al., 2018), whereas in animals, it produces intestinal changes and diarrheal episodes (Kolenda et al., 2015). Recently, Verotoxin-producing EAEC strains (O104: H4) identified from the German foodborne outbreak of 2011 have evidenced massive economic losses (Boll et al., 2020). Generally, EAEC is regarded as a heterogeneous pathogen; the pathogenicity of EAEC is described initially by its surface adherence to the intestinal mucosa, followed by biofilm formation and toxin release, which often ends up with an inflammatory response (Lima et al., 2018). The biofilm formation by EAEC is correlated well with the persistence of infection and recalcitrance to empirical antimicrobial treatment (Lin et al., 2017; Petro et al., 2020). This persistent colonization of EAEC leads to carrier status, enabling antibiotic pressure, which results in frightening levels of multi-drug resistance. Globally, drug resistance among EAEC strains toward first-line antibiotics (beta-lactams and fluoroquinolones) has been well evident (Lima et al., 2018). Moreover, multi-drug-resistant (MDR)-EAEC strains were recovered from food handlers (Oundo et al., 2008), diarrheal children, travelers’ diarrhea (Hebbelstrup Jensen et al., 2018), mangrove estuaries (Ghaderpour et al., 2015), and surface water (Canizalez-Roman et al., 2019).

Currently, with the advent of AMR crisis (Ghosh et al., 2019; Molnar, 2019) and limited discovery of newer antibiotics, the focus has now been shifted toward identifying effective alternative therapeutics (Haney et al., 2019). Many promising approaches have been reported for addressing bacterial resistance, which includes antibodies targeting specific pathogens, phage therapy, exolysins, endolysins including enzybiotics, vaccines, prebiotic and probiotic strains, antimicrobial peptides (AMPs), and phytochemicals (Ghosh et al., 2019). Of late, cationic AMPs have gained considerable attention concerning their antimicrobial and antibiofilm technology solutions (Haney et al., 2019). AMPs are evolutionarily conserved entities present in a wide range of organisms and have been heralded as a promising alternative to antibiotics (Mookherjee et al., 2020). The complex and multi-modal action of AMPs enables recalcitrance to develop perdurable microbial resistance that offers yet another advantage over conventional antibiotics (Kumar et al., 2018; Haney et al., 2019). However, long-chain peptides increase their cost for production and thereby investigation (de la Fuente-Núñez et al., 2015); hence, short-chain synthetic peptides have been attempted (de Zoysa et al., 2015). In particular, a 14-amino-acid residue of the Lactoferricin protein, namely, Lfcin (17–30), was proposed to exhibit broad-spectrum antimicrobial activities predominantly by way of membrane permeabilization compared to that of the naive milk protein, lactoferrin (Silva et al., 2017). Lfcin (17–30) has been identified to act against bacteria, fungi, amoeba, stimulants of bio-warfare agents, and even bacterial biofilms (van der Kraan et al., 2005; Xu et al., 2010; Sijbrandij et al., 2017; Acosta-Smith et al., 2018; Díaz-Godínez et al., 2019). Nevertheless, its use against MDR pathogens, such as EAEC, remains unrevealed, barring a few systematic studies against pathogens like *Klebsiella pneumoniae*, *Pseudomonas aeruginosa*, and *Acinetobacter baumannii* (Reyes-Cortes et al., 2016; Pollini et al., 2017). Even studies addressing the antimicrobial and antibiofilm potential of Lfcin (17–30) against MDR-EAEC are lacking.

Furthermore, *in vivo* manifestations of EAEC have been established in various mammalian models, which involve logistical, ethical, and budgetary constraints (Philipson et al., 2013; Kumar et al., 2016). Therefore, as an alternative *in vivo* model, *Galleria mellonella* larvae have recently been explored to evaluate the therapeutic potential of novel candidates including AMPs against various bacterial pathogens, including MDR-EAEC (McCloskey et al., 2019; Vergis et al., 2019). Moreover, the short life span of larvae and their ability to simulate humans while exploring pathogens of public health significance (Wojda, 2017; Cutuli et al., 2019; Blasco et al., 2020) extends their importance as an excellent *in vivo* model to screen novel therapeutics including AMPs. However, studies addressing *in vivo* antibacterial efficacy of Lfcin (17–30) against MDR-EAEC have never been explored in the *G. mellonella* larvae model. The objectives of the study were to assess *in vitro* antimicrobial as well as antibiofilm efficacy of Lfcin (17–30) against biofilm-forming MDR-EAEC strains and later to evaluate its antimicrobial efficacy in a *G. mellonella* larval model. Simultaneously, *in vitro* stability, safety, and mechanism of action of Lfcin (17–30) against MDR-EAEC were also evaluated to explore its possible utility as a therapeutic candidate.

## Materials and Methods

### Bacterial Strains

The typical EAEC isolates with NCBI GenBank accession numbers KY941936.1 (MDR 1), KY941937.1 (MDR 2), and KY941938.1 (MDR 3) maintained in the laboratory repository of Division of Veterinary Public Health, Indian Veterinary Research Institute, Izatnagar, were re-validated by PCR (Vijay et al., 2015), confirmed using HEp-2 adherence assay (Cravioto et al., 1979) and subjected to antibiotic susceptibility testing (Clinical and Laboratory Standards Institute [CLSI], 2018). *E. coli* ATCC 25922 was used as the quality control strain for antibiotic susceptibility testing.

### Antimicrobial Peptide

Lfcin (17–30) evaluated in this study was retrieved from BaAMPs (Di Luca et al., 2015), commercially synthesized from Shanghai Science Peptide Biological Technology, China, resuspended in phosphate-buffered saline (PBS; pH 7.40) with a final stock concentration of 10 mg/ml and stored at -20°C until further use (Supplementary Table S1). To compare the results of Lfcin (17–30), meropenem was used as an antibiotic treatment control throughout this study.

### Characterization of Lfcin (17–30)

Initially, characterization of Lfcin (17–30) was performed by determining its minimum inhibitory concentration (MIC), minimum bactericidal concentration (MBC), *in vitro* stability assays (high-end temperatures, the physiological concentration of cationic salts and proteases), and *in vitro* safety assays (sheep erythrocyte hemolysis and secondary cell line cytotoxicity) (Supplementary Files S1–S3). The membrane permeabilization effect of Lfcin (17–30) was assessed by flow cytometry, whereas outer and inner membrane permeability of MDR-EAEC strains treated with 1 × and 2 × MIC levels of Lfcin (17–30) was carried out based on the nitrocefin activity as well as the release of cytoplasmic β-galactosidase activity, respectively (Supplementary File S4). Further, Lfcin (17–30) was evaluated for its antibacterial activity against beneficial lactobacilli (*L. acidophilus* MTCC 10307 and *L. rhamnosus* MTCC 1408) (Supplementary File S5).

### *In vitro* Time-Kill Kinetics of MDR-EAEC With Lfcin (17–30)

The *in vitro* dose- and time-kill kinetics was assessed by co-incubating the log-phase cultures of each of MDR-EAEC isolates (ca. 1 × 10^7^ CFU/ml) in cation-adjusted Mueller Hinton (CA-MH) broth with MIC (1 × and 2 × ) of Lfcin (17–30) (Supplementary Information S6). The desired inoculum for each MDR-EAEC strain and Lfcin (17–30) suspended in CA-MH broth was as follows: Group I, 10^7^ CFU of MDR-EAEC (50 μl) with 1 × MIC of Lfcin (17–30) (50 μl); Group II, 10^7^ CFU of MDR-EAEC (50 μl) with 2 × MIC of Lfcin (17–30) (50 μl); Group III, 10^7^ CFU of MDR-EAEC (50 μl) with 10 μg/ml of Meropenem (50 μl); and Group IV, 10^7^ CFU of MDR-EAEC (50 μl) in CA-MH broth (50 μl). The MDR-EAEC counts were enumerated (Miles et al., 1938) at 0, 30, 60, 90, 120, 150, and 180 min, and 24, 48, and 72 h post co-incubation.

### *In vivo* Assays Using *G. mellonella* Model

The final instar of *G. mellonella* larvae was utilized for performing the *in vivo* assays (Morgan et al., 2014). The larvae (ca. 200–250 mg) inoculated with aliquots of MDR-EAEC suspensions (10 μl) by injection using Hamilton syringe (26 gauge) via the last right pro-leg were incubated at 37°C and the observations were noted. The larvae kept in a sterile environment were provided with *ad lib* food during the experiment.

The determined LD_50_ dose for each MDR-EAEC strain in the larvae was validated and employed further in the *in vivo* experiments to evaluate the antibacterial efficacy of Lfcin (17–30).

#### *In vivo* Antimicrobial Efficacy of Lfcin (17–30)

*Galleria mellonella* larvae were grouped 40 per group for *in vivo* antimicrobial testing of Lfcin (17–30) as follows: group I (MDR-EAEC infection control), groups II and III (infection + treatment groups), group IV (PBS control), and group V [Lfcin(17–30) control]. Optimized LD_50_ dose of MDR-EAEC (a cocktail of three strains) was administered to larvae of groups I to III, while MIC dose of Lfcin (17–30) and Meropenem was administered 3 h post-infection (pi) in groups II and III, respectively. Larval group IV was injected with sterile PBS, whereas MIC dose of Lfcin (17–30) was administered in group V.

The larvae were then observed for their survival rate, melanization (Supplementary Information S7), MDR-EAEC counts (Supplementary Information S8), hemocyte density (Supplementary Information S9) (Wand et al., 2013), lactate dehydrogenase (LDH) cytotoxicity assay (Supplementary Information S10) (Gibreel and Upton, 2013), and histopathology (Supplementary Information S11) (Perdoni et al., 2014), at an interval of 6 h till 24 h, followed by 24-h interval up to 120 h pi.

### Biofilm Formation by MDR-EAEC Isolates

The biofilm formation by MDR-EAEC strains was qualitatively assessed by Congo red binding assay and hydrophobicity index (Microbial adhesion to Solvents; MATS), while the time-dependent biofilm-forming ability was assessed in different media (Supplementary Information S12) as a semi-qualitative method.

#### Determination of Minimum Biofilm Eradication Concentration (MBEC) of Lfcin (17–30) Against MDR-EAEC Biofilm Formation

The MBEC of Lfcin (17–30) was determined against the preformed (48 h) MDR-EAEC biofilm (Ceri et al., 1999). MBEC value is the lowest concentration of Lfcin (17–30) that prevented the MDR-EAEC re-growth. This MBEC value of Lfcin (17–30) was further used to study the eradication of biofilm formed by MDR-EAEC strains.

### Inhibition and Elimination of MDR-EAEC Biofilm by Lfcin (17–30)

The efficacy of Lfcin (17–30) to inhibit and eliminate the biofilms of MDR-EAEC strains was evaluated by crystal violet (CV) staining as well as by the live/dead staining method (de Zoysa et al., 2015). Further, to quantify live and dead cells, the confocal images obtained were analyzed using Fiji ImageJ software ver. 1.51s (Schindelin et al., 2012). The varying bio-volume proportions of live and dead bacteria obtained after deducting the background score were plotted in intensity histogram and interpreted as a function of Red-Green intensity.

### Statistical Analysis

All the experiments were repeated individually and independently thrice; the data obtained were reflected as mean ± standard deviation for each assay using GraphPad Prism 5.01 software (GraphPad Software Inc., San Diego, CA). A one-way analysis of variance (ANOVA) with Bonferroni multiple comparison post-test was used to compare the differences observed in the *in vitro* cell line cytotoxicity assay and to analyze biofilm data, while paired two-tailed “*t*” test was used to analyze the antibacterial effect of Lfcin (17–30) on beneficial lactobacilli. A two-way (repeated measures) ANOVA with Bonferroni multiple comparison post-test was used to analyze the *in vitro* and *in vivo* time-dependent antimicrobial assays [inner and outer membrane permeability and killing kinetic assays of Lfcin (17–30), *in vivo* bacterial burden, melanization, hemocyte enumeration, and LDH assays]. The probit regression model was used to determine the LD_50_ dose of MDR-EAEC strains, while the log-rank (Mantel-Cox) test and log-rank test for trends were used for *in vivo G. mellonella* larval survival curves. A *P* value of ≤0.01 was considered highly significant, while a *P* value ≤0.05 was considered statistically significant.

## Results

In this study, all the three typical MDR-EAEC wild strains employed were found to be resistant to four or more classes of antibiotics and were ESBL producers (Supplementary Table S2).

### Characterization of Lfcin (17–30)

The MIC and MBC values of Lfcin (17–30) against the tested MDR-EAEC strains were found to be equal (32 μM).

Lfcin (17–30) was found stable at high-end temperatures (70 and 90°C; Supplementary Table S3A), protease (proteinase-K and trypsin; Supplementary Table S3B), and physiological concentration of cationic salts (150 mM NaCl and 2 mM MgCl_2_; Supplementary Table S3C), as MIC and MBC values of Lfcin (17–30) were found unaltered.

Lfcin (17–30) was found to be non-hemolytic at lower (1 × and 2 × MIC) concentrations; however, at 4 × MIC, minimal hemolysis (<5%) was noted (Supplementary Table S4). Similarly, Lfcin (17–30) marginally reduced the viability of secondary cell lines (HEp-2 and RAW 264.7) tested in a concentration-dependent manner. At lower concentrations (1 × and 2 × ), remarkable morphological changes were not evident; however, at 4 × MIC, moderate to higher cytotoxicity was evident in HEp-2 and RAW 264.7 cells (Supplementary Figure S1).

Also, a non-significant (*P* > 0.05) antimicrobial effect was observed against beneficial lactobacilli (*L. acidophilus* and *L. rhamnosus*; Supplementary Figure S2), suggesting the safety of Lfcin (17–30) against beneficial lactobacilli.

**FIGURE 1:**
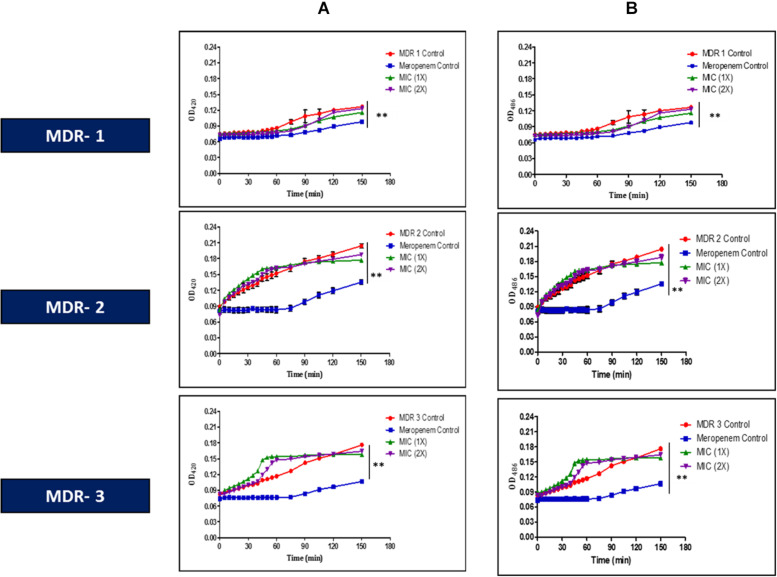
*In vitro* membrane permeability assay of Lfcin (17–30) in MDR-EAEC strains. MIC (1 × and 2 × ) concentrations of Lfcin (17–30) against MDR-EAEC strains employing ONPG **(A)** and nitrocefin **(B)**, expressed as a function of time at 37°C. Hydrolysis of ONPG by β-galactosidase was used to monitor inner membrane permeabilization by absorbance at 420 nm, while hydrolysis of nitrocefin by β-lactamase was used to monitor outer membrane permeabilization by absorbance at 486 nm (***P < 0.001; **P < 0.01).

Lfcin (17–30), at MIC (1 × ), exhibited remarkable damage to the cell membrane of MDR-EAEC strains (PI-positive cells >50%), as evidenced by flow cytometry (Supplementary Figure S3). Moreover, Lfcin (17–30) permeated the inner as well as outer membrane of all the MDR-EAEC strains in the uptake of ONPG (Figure 1A) and nitrocefin (Figure 1B), respectively, in a concentration- and time-dependent manner. Moreover, the onset and progress of both inner and outer membrane permeabilization by Lfcin (17–30) was significantly quicker (*P* < 0.01) than the antibiotic control, meropenem.

### *In vitro* Time-Kill Kinetics of MDR-EAEC With Lfcin (17–30)

**FIGURE 2:**
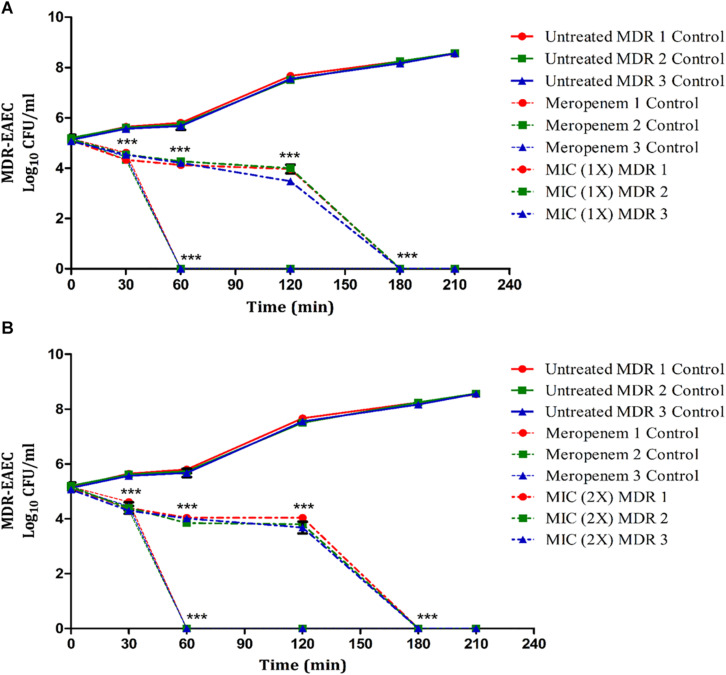
Dose- and time-dependent time-kill assay of MDR-EAEC isolates co-cultured with Lfcin (17–30). MDR-EAEC isolates (*n* = 3) were co-cultured with 1 × MIC of Lfcin (17–30) **(A)** and 2 × MIC of Lfcin (17–30) **(B)** in CA-MH broth at 37°C under static conditions with respective controls of MDR-EAEC isolates (untreated and meropenem-treated). Data expressed as the mean ± standard deviation (log_10_CFU/ml) of three independent experiments (***P < 0.001).

The antimicrobial effect of Lfcin (17–30) at 1 × and 2 × MIC was highly significant (*P* < 0.001) at 30 min post-coincubation (Figure 2). Further, none of the MDR-EAEC isolates exhibited any visible growth at 180 min post-coincubation. In contrast, an increased growth pattern was observed in the untreated control group at 30, 60, 90, 120, 150, and 180 min post-coincubation (Figure 2). Additionally, a highly significant (*P* < 0.001) reduction in bacterial counts was observed in the meropenem treatment group at 30 min post-coincubation, and after 60 min post-coincubation, complete inhibition of MDR-EAEC isolates was observed (Figure 2). Since no significant difference (*P* > 0.05) was observed between the MIC and MBC values of Lfcin (17–30) in inhibiting all three MDR-EAEC strains (Figure 2), further *in vivo* studies in *G. mellonella* larvae were carried out using the MIC value of Lfcin (17–30).

### LD_50_ of MDR-EAEC Strains in the *G. mellonella* Larval Model

While optimizing the LD_50_ dose of MDR-EAEC, a concentration-dependent mortality rate was observed in the *G. mellonella* larvae survival plots (Supplementary Figure S4). An inoculum of 10^6^ CFU/larvae was determined as the optimized LD_50_ dose of MDR-EAEC strains (Supplementary Figure S4).

### *In vivo* Antimicrobial Efficacy of Lfcin (17–30)

**FIGURE 3:**
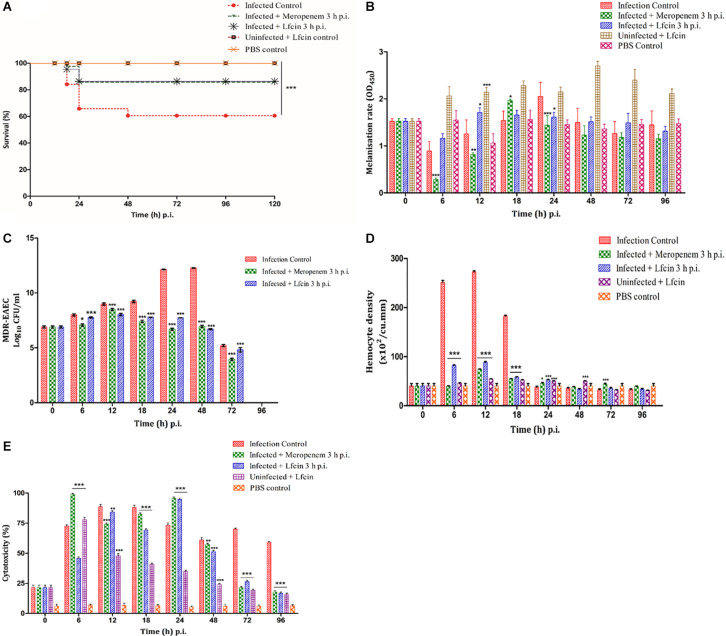
*In vivo* assays using *G. mellonella* model. Survival plot **(A)**, melanization rate **(B)**, MDR-EAEC counts **(C)**, hemocyte density **(D)**, and LDH cytotoxicity assay **(E)** of *G. mellonella* larvae infected with LD_50_ dose (10^6^ CFU) of MDR-EAEC strains and treated with MIC (1 × ) of Lfcin (17–30) 3 h pi. MDR-EAEC-induced infection was treated with MIC of Lfcin (17–30), keeping respective controls [infection control, meropenem treatment, Lfcin (17–30) control, PBS control]. Data expressed as the mean ± standard deviation of three independent experiments (*P < 0.05; **P < 0.01; ***P < 0.001). Melanization rate was assessed by absorbance monitored at 450 nm, MDR-EAEC counts as log_10_CFU/ml of hemolymph on EMB agar plates supplemented with ampicillin (100 μg/plate), hemocyte density as cells/ml of hemolymph, and LDH cytotoxicity assay as the cytotoxicity (%) of larval hemolymph.

In the MDR-EAEC infection control larval group, a survival rate of 52.50% was observed, while an enhanced survival rate (85%) was observed in the meropenem-treated group up to 120 h pi (Figure 3A). Further improved survival rate (90%) was noticed in the Lfcin (17–30)-treated infected larval group that corresponded to a significant log-rank Mantel–Cox test (*P* < 0.001) and log-rank test for trend (*P* < 0.05) (Figure 3A). All the uninfected larval control groups [PBS control and Lfcin (17–30) control] were found to be healthy with a 100% survival rate up to 120 h pi (Figure 3A).

### Melanization Assay

The rate of melanization was lower in the MDR-EAEC infection control larval group at 6 h pi, increased thereafter and reached its peak at 24 h pi, and was found to reduce at 48 h pi (Figure 3B). In the meropenem-treated infected group, the melanization rate was found to be lower at 6 h pi, peaked at 18 h pi, and gradually declined thereafter in a highly significant (*P* < 0.001) manner (Figure 3B). However, in the infected group treated with Lfcin (17–30), melanization was found to increase at 12 to 24 h pi; thereafter, the intensity of melanization was gradually declined (Figure 3B). In uninoculated larval groups treated with Lfcin (17–30), a slight increase in the melanization rate was observed at 6 h pi; thereafter, the melanization rate was observed at an increased intensity at 12 to 96 h pi (Figure 3B).

### Enumeration of MDR-EAEC Counts

A significant reduction (*P* < 0.001) of MDR-EAEC counts was observed in the infected larval groups treated with Lfcin (17–30) at 24 h pi (mean 4.40 log reduction) and 48 h pi (mean 5.50 log reduction) as compared to the infected control group (Figure 3C). Further, MDR-EAEC was not detected in the hemolymph obtained from the uninoculated larval groups until 96 h pi (Figure 3C).

### Enumeration of Hemocytes

The inoculated and the treatment groups of *G. mellonella* larvae revealed significantly (*P* < 0.001) increased hemocyte density at 6 h pi; later, hemocyte density peaked by 12 and reduced thereafter in a significant (*P* < 0.001) manner (Figure 3D); however, a significant difference (*P* > 0.05) in the hemocyte density was not noticed between all the larval groups at 72 to 96 h pi (Figure 3D).

### LDH Assay

In the infected control group, LDH cytotoxicity increased in a highly significant manner (*P* < 0.001) at 6 h pi, peaked at 12 to 18 h pi, and retained cytotoxicity up to 96 h pi (Figure 3E). A significant (*P* < 0.001) increase in the cytotoxicity was noticed in the Lfcin (17–30)-treated infected larval group at 6 h pi and the cytotoxicity remained increased up to 48 h pi and thereafter a progressive reduction in cytotoxicity was observed (Figure 3E). Almost similar observations were noted in the infected group treated with meropenem (Figure 3E). In the uninoculated larval group treated with Lfcin (17–30), significantly increased cytotoxicity was observed at 6 to 18 h pi, and later it declined progressively (Figure 3E).

### Histopathological Examination

All the groups, except for the infected control group, revealed no histopathological alteration at 6 and 12 h pi, while hemocytes were sparsely distributed with bare melanization at 12 to 18 h pi. However, hemocytes were more pronounced in the sub-cuticular area of the infected control group, at 12 to 18 h pi exhibiting bacterial phagocytic reaction with pronounced melanization and EAEC load surrounding the tubular organs. Further, cross-sections of the infected control larvae revealed clusters of hemocytes in the sub-cuticular area at 18 h pi exhibiting bacterial phagocytosis, which was reflected as finely stippled blue dots along with melanization; besides, bacterial load was observed around tubular organs. Nevertheless, Lfcin (17–30)-treated infected larvae and uninoculated control groups (groups IV and V) appeared healthy with sparse distribution of hemocytes exhibiting minimal melanization.

The inoculated control larvae exhibited increased pathological abnormalities at 24 and 48 h pi, which declined progressively at 72 h pi, while histopathological changes were mild to moderate in the Lfcin (17–30)-treated group at 24 h pi (Supplementary Figure S5), which declined progressively at later time points (48 and 72 h pi). Interestingly, pathological changes could not be appreciated in inoculated groups treated with Lfcin (17–30), the uninoculated control group, and the PBS control group.

### Biofilm Formation by MDR-EAEC Isolates

Congo red binding assay revealed moderate to strong biofilm formation by MDR-2 and MDR-3 EAEC strains, while MDR-1 revealed weak biofilm formation (Supplementary Figure S6). Moreover, the hydrophobicity index indicated that all three MDR-EAEC strains employed were strongly basic and weakly acidic (Supplementary Figure S7). Further, the biofilm formation was highly significant (*P* < 0.001) in Dulbecco’s minimal essential medium (DMEM) containing 0.45% D-glucose on polystyrene surface at 48 h of incubation (Supplementary Figure S8). All the MDR-EAEC strains employed in this study were found to be moderate biofilm producers by microtiter plate assay (Wakimoto et al., 2004), as evidenced by OD_595_ values ranging from 0.375 to 0.537.

### Determination of MBEC of Lfcin (17–30)

The MBEC values observed for Lfcin (17–30) were equal to the MBC values (32 μM) of Lfcin (17–30) against biofilm formed by the MDR-EAEC strains.

### *In vitro* Efficacy of Lfcin (17–30) Against MDR-EAEC Biofilm

**FIGURE 4:**
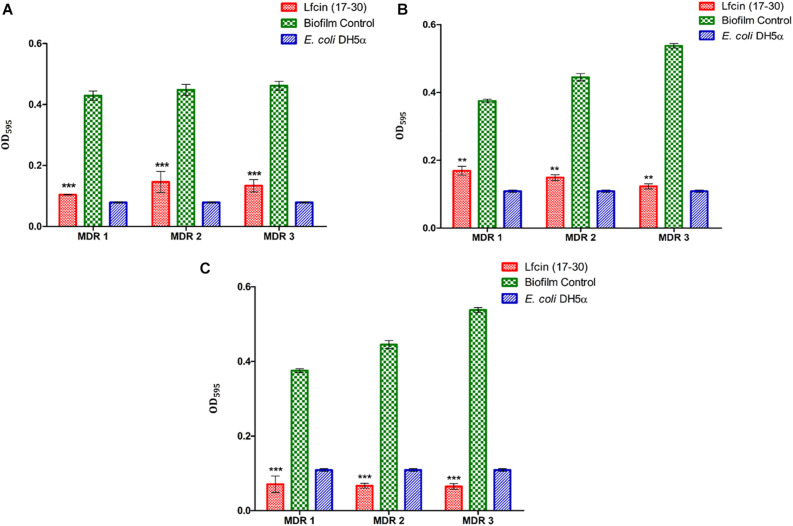
Inhibition and eradication of MDR-EAEC biofilm by Lfcin (17–30) by CV staining. Inhibition of MDR-EAEC biofilm by Lfcin (17–30) at 24 h **(A)**, 48 h **(B)**, and effect of treating MDR-EAEC preformed biofilms (48 h) with Lfcin (17–30) for an additional 24 h **(C)** by CV staining. Error bars indicate the standard deviation between strains. Control bars indicate corresponding MDR-EAEC strains (biofilm control) and *E. coli* DH5α biofilms, respectively, without AMP (***P < 0.001; **P < 0.01).

As evidenced by CV staining, the biofilm biomass of MDR-EAEC strains was reduced significantly at 24 h (*P* < 0.001) and 48 h (*P* < 0.01) on treatment with Lfcin (17–30) as compared to their respective controls (Figures 4A,B). Likewise, confocal microscopy also revealed a highly significant reduction in the biofilm biomass formed by MDR-EAEC isolates; however, significant inhibition in the biofilm biomass was observed at 24 h (*P* < 0.05) (Supplementary Figure S9) and 48 h (*P* < 0.01) (Supplementary Figure S9).

Lfcin (17–30) also eliminated the preformed biofilm of MDR-EAEC strains significantly (*P* < 0.001) as shown by CV staining (Figure 4C) and confocal microscopy (Supplementary Figure S10).

## Discussion

The emergence of multi-drug resistance as a result of “selection pressure” enables the pathogens to tolerate various antibiotic classes suggested for empirical therapy (Davies and Davies, 2010). With the limited availability of effective antibiotics against drug-resistant pathogens, the focus has now been targeted toward alternative strategies for treating infections. Of late, owing to their multi-faceted antimicrobial, antibiofilm, as well as immunomodulatory potential, studies employing AMPs have attracted additional momentum. Besides, AMPs have not been widely reported to gain resistance, enabling them to be chosen for treating drug-resistant, chronic, as well as persistent infections (Ghosh et al., 2019). EAEC causes chronic and persistent diarrhea due to its inherent biofilm-forming capability, which often ends up damaging the intestinal epithelium of human infants and young animals (Lima et al., 2018). Of late, emerging trends in AMR, particularly resurgence of MDR-EAEC and its rapid dissemination, have been reported from various sources (Kong et al., 2015; Lima et al., 2018), which represent an alarming public health threat and clinical challenge.

In this study, we attempted to assess the *in vitro* antimicrobial as well as antibiofilm efficacy of the Lfcin (17–30), a short-chain cationic AMP, from the regularly updated biofilm active AMPs (BaAMPs) database (Xu et al., 2010) against biofilm-forming MDR-EAEC field isolates and later to evaluate its antimicrobial efficacy in a *G. mellonella* larval model. Short-chain peptides (12–50 amino acids), preferably with cationic amino acids and a high proportion of hydrophobic residues (∼50%), were testified to be operative against biofilm-forming bacterial pathogens (de la Fuente-Núñez et al., 2015). Lfcin (17–30), a tetradecapeptide, has been reported to produce depolarization, loss of cytoplasmic inner membrane integrity, and pH gradient, thereby exerting a bactericidal effect, particularly on *E. coli* and also affect intra-cellular activities (Haukland and Vorland, 2001).

Despite having an excellent antibacterial activity, the clinical translation of AMPs involves certain limitations such as thermostability, proteolytic degradation, and salt inactivation within the body (Mohamed et al., 2016). In the present study, Lfcin (17–30) could withstand high-end temperatures (70 and 90°C). Generally, such high-end temperatures are often required for food processing, especially during the pelleting process to reduce the risk of microbial contaminants (Ebbensgaard et al., 2015). Based on this result, the intended incorporation of Lfcin (17–30) in the feed supplement is possible. Further, AMPs could be susceptible to proteolytic degradation by bacterial proteases together with gastrointestinal enzymes and serum proteases; however, in this study, Lfcin (17–30) was found to be protease-stable. Also, Lfcin (17–30) was found to be stable in the physiological concentration of cationic salts. Normally, the stability of AMP is highly dependent on amino acid residues such as tryptophan and arginine, which have previously shown to improve the antimicrobial activity of AMP under challenging salt conditions (Mohamed et al., 2016).

Cationic AMPs, like Lfcin (17–30), as employed in this study, may exhibit durable electrostatic interactions with the negatively charged phospholipids on the outer leaflet of the bacterial cell membrane and the least against eukaryotic cell membranes (Kumar et al., 2018). In our study, negligible hemolysis was caused by Lfcin (17–30) in sheep erythrocytes; nevertheless, the obtained findings need to be reasoned in the light of other cytotoxicity assays before ascertaining its utility as a potential therapeutic candidate. Additionally, Lfcin (17–30) reduced the viability of secondary cell lines (HEp-2 and RAW 264.7) in a concentration-dependent manner. Besides, typical cytotoxic effects such as detachment of confluent monolayer and cytoplasmic vacuolization were noticeably witnessed at higher concentrations (4 × MIC) of peptide, which might be attributed to its mechanism of action; however, the exact mechanism by which cytotoxicity varied was not understood absolutely (Vaucher et al., 2010). Furthermore, as part of the innate immune mechanism, AMPs may shape the composition of beneficial microbiota in maintaining intestinal homeostasis and assist in increasing the epithelial cells to avert bacterial uptake. Regardless of treatment with Lfcin (17–30), a non-significant effect was noticed on the tested beneficial lactobacilli, thereby proving its safety toward beneficial gut lactobacilli (Muniz et al., 2012).

The time-kill kinetic assay of Lfcin (17–30) exhibited a complete elimination of MDR-EAEC in 180 min, while similar inhibition was observed in meropenem after 60 min. Such complete bacterial inhibition by cationic peptides represents better treatment outcomes over antibiotics. Apart from the density of positively charged residues and an optimal balance between the hydrophilic and hydrophobic peptide surfaces, amphiphilic peptide confirmation is also responsible for the ability of AMPs to kill MDR-EAEC by disrupting bacterial membranes (Kumar et al., 2018). The membrane lipid-bilayer partition ability and optimum hydrophobicity might have led to the membrane damaging ability of Lfcin (17–30), as witnessed in flow cytometry, nitrocefin, and β-galactosidase activity (Marri et al., 1996; Epand et al., 2010). The antimicrobial activity of Lfcin (17–30) might be the result of its ability to stimulate the unfettered passage of small polar molecules through the cytoplasmic membrane. The time-kill kinetic assay in connection with the membrane permeabilization assay suggested the role of compromised and disrupted membrane integrity and pore formation as the main mechanism of cell killing by Lfcin (17–30).

High-throughput screening of antimicrobial agents require reliable and ergonomic *in vivo* laboratory models that could simulate humans. Though mammalian models are widely exploited, the budgetary, ethical, as well as logistic complications create hurdle in large-scale settings. These concerns in lab animal welfare warrants the possibility of utilizing invertebrate models in expediting the efficacy of antimicrobial agents for preliminary *in vivo* screening to reduce the candidate molecules for its further evaluation in ethical mammalian models. Having shared the innate immune response to the microbes (Wojda, 2017; Cutuli et al., 2019; Blasco et al., 2020), insects could be selected as suitable alternative models for extrapolating *in vitro* laboratory findings for probing the potential of Lfcin (17–30) against MDR-EAEC strains. The innate immune system of *G. mellonella* comprises a progressive cellular as well as humoral response compared to other existing invertebrate models. Although earlier reports regarding the *in vivo* efficacy of various antimicrobial agents against pathogens such as carbapenem-resistant Enterobacteriaceae, MRSA, *P. aeruginosa*, and *K. pneumonia* have explored the *G. mellonella* larvae model (Gibreel and Upton, 2013; Betts et al., 2014; Benthall et al., 2015; Cutuli et al., 2019), studies addressing the use of AMPs against such pathogens are scarcely documented in the literature. In the present study, dose-dependent mortality was reported in the larvae wherein the survival decreased with an increasing inoculum of MDR-EAEC. The determined LD_50_ dose (1 × 10^6^ CFU/larvae) differed from an earlier study (1.11 × 10^4^ CFU/larvae) (Jønsson et al., 2017), which could probably be due to varying levels in the virulence of EAEC strains.

The efficacy of AMPs against MDR-EAEC was assessed in comparison with an effective antibiotic, meropenem. The significant survival rate observed among meropenem-treated larvae, as reported in earlier studies (Hill et al., 2014; Benthall et al., 2015), might be due to the varied pharmacokinetic factors as compared to human, with a better antibiotic bioavailability in the larvae or with the synergistic effect of carbapenem with the natural AMPs present in the hemolymph. A significant increase observed in the survival rate of MDR-EAEC-infected larvae treated with Lfcin (17–30) in this study correlates with the earlier findings wherein different antimicrobials were tried against MRSA, *A. baumanii*, *F. tularensis*, and *B. multivora* (Ahmad et al., 2010; Brackman et al., 2011; Hill et al., 2014). The complete survival along with the lack of melanization of uninfected larval groups suggests the *in vivo* safety of Lfcin (17–30) tested and that the data need to be correlated with *in vitro* cytotoxicity assays. The result of the present study suggests that Lfcin (17–30) has an almost identical antibacterial potential to or a far better antibacterial potential than the antibiotic control used in this study; however, the exact AMP–host interaction remains uncertain.

The significant reduction in MDR-EAEC counts recovered from the infected larval hemolymph treated with Lfcin (17–30) over 24 and 48 h pi might be due to the bactericidal effect of AMP and/or metabolites produced during melanization (Hornsey and Wareham, 2011). The activation of the prophenoloxidase (PPO) cascade pathway in invertebrates is responsible for melanization wherein enhanced superoxide production in the larvae leads to the release of host AMPs, which in turn might be responsible for the clearance of pathogenic microbes (Wand et al., 2011). The bacterial clearance observed at 96 h pi could be achieved either by hemocyte-mediated aggregation or bacterial phagocytosis, which would have resulted in the secretion of larval AMPs leading to hemocyte degradation and larval melanization (Zhao et al., 2007; Desbois and Coote, 2011).

MDR-EAEC-stimulated hemocytes might successfully phagocytose the bacterial agents during the initial phase of infection at 6 to 18 h pi that has correlated fairly with the findings of MDR-EAEC enumeration wherein no significant difference in the MDR-EAEC counts could be noticed between the infection control group and the Lfcin (17–30)- and meropenem-treated infected larval groups. However, the MDR-EAEC, which evades phagocytosis and is located within the hemocytes, starts to replicate 24 h pi in the larvae, which is evident in the bacterial enumeration assay. A reduction in the circulating hemocytes noticed in all the larval groups at 72 and 96 h pi might be the result of cytotoxicity of MDR-EAEC strains on the larval cells (Amorim-Vaz et al., 2015). This depletion in hemocyte density could probably be ascribed to the infected hemocyte degradation and/or hemocyte sequestration in the nodules. However, upon any bacterial infection, the depletion of circulating hemocytes triggers the release of PPO components into the hemolymph, leading to the activation of phenoloxidase (PO). The activity of PO induces the formation of quinones and melanin that act as key components for defense reactions against the invading microbes (Barnoy et al., 2017).

Further, the results of melanization assay correlated fairly well with the hemocyte enumeration assay wherein with the decrease in hemocyte density, an increase in melanization intensity (12–24 h pi) was observed. The elevated melanization intensity observed in treatment groups infected with MDR-EAEC (12–24 h pi) could be interrelated with the activation of PO melanization cascade by stimulating hemocytes, which in turn might have led to the secretion of AMP within the fat body of insects, which is analogous to a liver of mammals (Barnoy et al., 2017). This would have activated specific signaling pathways leading to the generation of reactive oxygen species that might have destroyed the invading microbes (Desbois and Coote, 2011). It could also be inferred that Lfcin (17–30) improved the immunomodulation of larvae, as the melanization intensity was retained up to a later time point (96 h pi) in both uninfected and Lfcin (17–30)-treated infected larval groups. These observations suggest Lfcin (17–30) as a potential candidate for the development and re-purposing of drugs (Kumar et al., 2018).

Upon inoculation of MDR-EAEC, an elevated LDH production was observed in the inoculated larval group due to the increase in damaged and apoptotic host cells. The increasing trend in LDH production was observed in the meropenem-treated group wherein earlier studies reported the use of ampicillin as an effective therapeutic candidate for *P. aeruginosa* infection in *G. mellonella* larvae (Benthall et al., 2015). An initial increase in LDH production in all the inoculated groups could be attributed to the early host cell damage caused by pathogen before EAEC interacted with AMP. Besides, the minor trauma, while infecting the larvae and/or while administering the AMP could not be ignored, for the initial elevation of LDH. Furthermore, had Lfcin (17–30) been toxic to the larvae, the survival rate would have been reduced in all the groups treated with Lfcin (17–30), suggesting that Lfcin (17–30) was not cytotoxic to the larval cells.

Histopathological examination of the whole larvae was performed to divulge the sequence of events with host–pathogen interaction, pathogen migration, as well as recruitment of hemocytes (Pereira et al., 2015). It was observed that in the control infected group, the phagocytosis of MDR-EAEC mediated by hemocytes occurs promptly with the enrolment of hemocytes toward the heart region, where the hemocytes bind to the muscular architecture of heart and lead to phagocytosis of MDR-EAEC during 24 and 48 h pi. Later at 72 h pi, the hemocytes were evident in the heart and surrounding organs (pericardial cells and fat body), which, in turn, might have resulted in reducing the load of MDR-EAEC, melanization rate, as well as the circulating hemocyte density. Such sessile nature of larval hemocytes plays a vital role in controlling EAEC infection and pathogen recruitment.

Further, this fact reinforces the postulation that a coordinated interaction between the open circulatory system and the innate immune mechanism of the larvae is pivotal for operative immune responses (Lu et al., 2014; Pereira et al., 2015; Sheehan and Kavanagh, 2018). Nevertheless, a mild bacterial accumulation observed around the organelle without the pronounced aggregation of hemocytes or melanization observed at 24 and 48 h pi in the inoculated larval groups treated with Lfcin (17–30) and meropenem could serve as an indication of hemocyte-mediated phagocytosis that sequestered the pathogen within the internal organs and phagocytose them. None of the healthy larval controls exhibited appreciable histopathological changes with AMP treatment, which suggested the *in vivo* safety potential of Lfcin (17–30) in the *G. mellonella* larval model. The histopathology of the larval model was found closely interrelated with the MDR-EAEC burden, with estimation of various immune markers (melanization, hemocyte density), and with the *in vitro* time-kill kinetic assays. Additionally, it was concluded that those factors permitting the larval survival could be well pertinent to similar infections in humans (Sheehan and Kavanagh, 2018; Blasco et al., 2020).

Further, regarding microbial biofilm, nearly 80% of microbial infections in the living system involve biofilm formation (Batoni et al., 2016). Biofilm constitutes a syntrophic association of microbes enclosed within extracellular polymeric substance matrix that prevents microorganisms from adverse external influences, particularly antimicrobial agents (Lin et al., 2017; Petro et al., 2020). The adhesion of MDR-EAEC strains by MATS in this study was found to be comparatively higher to the tested monopolar solvent, chloroform, than to the apolar solvent, n-hexadecane, which bears almost identical van der Waal’s properties. Hence, the tested MDR-EAEC strains were deduced as strongly basic and weakly acidic. MDR-EAEC isolates employed in this study revealed significant hydrodynamic growth in DMEM supplemented with 0.45% D-glucose on the PS surface at 48 h. Besides, based on the grading criterion, moderate biofilm-forming ability (absorbance at 595 nm: 0.325–0.648) was observed for all the MDR-EAEC strains tested (Vijay et al., 2015).

The CV staining and confocal microscopy-based live/dead cell quantification revealed a significant biofilm biomass inhibition of MDR-EAEC strains by Lfcin (17–30) at 24 and 48 h. It could be speculated that for such initial inhibition of biofilm formation, Lfcin (17–30) could act as a coating agent covering either the surface of bacteria or biomaterials or both. The similar concentrations obtained for MBEC as well as MBC could be due to the moderate biofilm-forming ability of bacterial strains tested. Further, the elimination of preformed MDR-EAEC biofilms by peptides could be attained either by way of its direct antibacterial activity or detachment of live bacteria from the biofilms (Mataraci and Dosler, 2012; Silva et al., 2016; Zhang et al., 2016). However, the detachment and release of live bacterial cells from the preformed microbial biofilms by cationic peptides have not yet been documented.

The AMPs may be subjected to further clinical trials in suitable mammalian models before being translated as effective therapeutic candidates in either humans or animals. Many of the identified AMPs reported to have failed prior to or even during clinical trials. To date, only seven peptides were approved by the Food and Drug Administration (FDA) for therapeutic purpose. Though peptides are widely reported to treat skin infections, wounds, and pink eye as topical agents, some of them are also administered parentrally by oral route and direct injection (Chen and Lu, 2020). Moreover, conjugation of peptides with active molecules such as antibodies and/or nanoparticles, computational predictions, and high-throughput screening could be employed to make AMPs less toxic for target species while maintaining or improving their efficacy to eliminate pathogens, and further clinical studies in this regard is highly necessitated.

## Conclusion

The antimicrobial and antibiofilm potential of Lfcin (17–30) was evaluated for the first time against the MDR-EAEC field isolates. Interestingly, Lfcin (17–30) was found to be stable (at high-end temperatures, proteases, and cationic salts), safe for eukaryotic cells, and beneficial to lactobacilli. Additionally, Lfcin (17–30) elicited a pronounced immunomodulatory effect and proved to be non-cytotoxic to the larval cells; overall, Lfcin (17–30) was found to be highly efficacious in the *G. mellonella*-EAEC infection model. Additionally, Lfcin (17–30) could inhibit the initial biofilm formation as well as eliminate the preformed biofilm of MDR-EAEC strains. Further studies using Lfcin (17–30) in mammalian models (mice/piglets) are warranted. Moreover, for the judicious application of Lfcin (17–30), the AMP can be coupled with targeted drug-delivery systems to prove its efficacy against MDR-EAEC in the infected mice or piglet models.

## Data Availability Statement

The raw data supporting the conclusions of this article will be made available by the authors, without undue reservation.

## Author Contributions

DR, SM, and SB contributed to the conception and design of the study. JV and RP organized the experiments. MK and SR performed the statistical analysis. JV and MK wrote the first draft of the manuscript. JV, RP, MK, NK, and SR wrote sections of the manuscript. DR, SM, NK, and SB edited the manuscript. All authors contributed to manuscript revision and read and approved the submitted version.

## Conflict of Interest

The authors declare that the research was conducted in the absence of any commercial or financial relationships that could be construed as a potential conflict of interest.
