# Supplementary material for: Exploiting Lactoferricin (17–30) as a Potential Antimicrobial and Antibiofilm Candidate Against Multi-Drug-Resistant Enteroaggregative Escherichia coli
Source: Front Microbiol. 2020 Sep 18;11:575917. doi: 10.3389/fmicb.2020.575917 (PMC7531601; doi:10.3389/fmicb.2020.575917)
Supplement: Supplementary file 1 [file Data_Sheet_1.doc]

***ELECTRONIC SUPPLEMENTARY INFORMATION***

**S1. Determination of Minimum Inhibitory Concentration (MIC) and Minimum Bactericidal Concentration (MBC) of Lfcin (17-30) against MDR-EAEC strains**

The MIC and MBC values of Lfcin (17-30) against ‘planktonic’ MDR-EAEC cells were determined using the micro-broth dilution method [1]. The lowest dilution of Lfcin (17-30) revealing no visible growth was designated as MIC, while, MBC value for Lfcin (17-30) was determined at the end of the 24 h incubation period. In brief, 10 µL of seeded inoculum was drawn from each well, having no visible growth and were placed on Eosin Methylene Blue agar (HiMedia, Mumbai, India). The lowest concentration that produced at least 99.9% killing of the initial inoculum of planktonic cells was considered as the MBC.

**S2. Stability studies of Lfcin (17-30)**

To explore the utility of Lfcin (17-30) as a therapeutic molecule, *in vitro* stability was evaluated by exposing it to high-end temperatures, proteases as well as the physiological concentration of salts. In brief, the effect of temperature was investigated by incubating Lfcin (17-30) at 70^0^C and 90^0^C for 5, 15, and 30 min followed by measuring the MIC value [2]. The effect of proteases (trypsin and proteinase-K) on the antimicrobial activity of Lfcin (17-30) [Protease: AMP- 1:100 (w/w)] was investigated by incubating with the respective protease at 37^0^C for 30 sec, 2, 5, 15 and 30 min, followed by inactivating the enzyme at 90^0^ C for 10 min and measuring the MIC value [2]. The stability of Lfcin (17-30) in the presence of high salt concentrations (150 mM and 2 mM MgCl_2_) was estimated in regular MH broth [2]. Appropriate controls were included in each experiment.

**S3. Safety studies of Lfcin (17-30)**

To determine the safety of Lfcin (17-30) on host cells, a haemolytic assay using sheep erythrocytes as well as cytotoxicity assay using secondary cell lines were performed with 1X, 2X and 4X MICs of Lfcin (17-30), respectively. In brief, haemolytic assay was performed [2], wherein the haemolytic activity of Lfcin (17-30) was tested by determining the extent of haemoglobin release from sheep erythrocytes (10%). Further, the safety aspect of Lfcin (17-30) was evaluated on secondary cell line *viz*., human epithelioma cell line (HEp-2), and murine macrophage cell line (RAW 264.7) by performing MTT [3-(4,5-dimethylthiazole-2-yl)-2,5-diphenyl tetrazolium bromide] assay [3].

Besides, the safety aspect of Lfcin (17-30) against beneficial gut lactobacilli was also investigated. Briefly, Mann Rogosa Sharpe (MRS) broth medium (100 µl) containing Lfcin (17-30) (1X MIC) was inoculated with 100 µl of each beneficial gut lactobacilli (*Lactobacillus rhamnosus* and *Lactobacillus acidophilus* ca. 1x10^7^ CFU/ml) in 96-well microtiter plates. Each plate included a positive growth control (untreated commensal flora) and negative control (MRS broth). After incubation at 37^0^C for 48 h, the antibacterial effect of AMP on commensal lactobacilli was measured by observing the absorbance at 600 nm (Thermo Scientific Multiskan GO) as well as drawing two 10 µl samples from each well and plating them onto MRS agar plates [4].

**S4. Mechanism of action of Lfcin (17-30) on MDR-EAEC strains**

To determine the mechanism of action of Lfcin (17-30) on MDR-EAEC strains, initially membrane permeability effect of Lfcin (17-30) on MDR-EAEC was studied by incubating the AMP-treated bacterial cells with the membrane impermeant dye, propidium iodide (PI) and further analyzed by flow cytometry (FACS) [5]. Also, the inner and outer membrane permeability of MDR-EAEC isolates treated with MIC (1X and 2X) values of Lfcin (17-30) was performed to measure the release of cytoplasmic β-galactosidase [6] and nitrocefin activity [7], respectively.

**S5. *In vitro* antibacterial effect of Lfcin (17-30) on commensal gut flora**

Briefly, Mann Rogosa Sharpe (MRS) broth medium (100 µl) containing 1X MIC of Lfcin (17-30) was inoculated with 100 µl of each commensal bacteria (ca. 1x10^7^ CFU/ml) in 96-well microtiter plates. Each plate included a positive growth control (untreated commensal flora) and negative control (MRS broth). Subsequent to the incubation at 37^0^C for 48 h, Lfcin (17-30) was tested for the effect on commensal flora by observing the absorbance at 600 nm (Thermo Scientific Multiskan GO) as well as drawing two 10 µl samples from each well and plating the samples onto MRS agar plates [4].

**S6. *In vitro* dose and time-dependent growth kinetics of MDR-EAEC with Lfcin (17-30)**

The desired bacterial numbers for each MDR-EAEC isolate and Lfcin (17-30) were suspended in CA-MH broth as follows: Group I, 10^7^ CFU of MDR- EAEC (50 µl) with 1X MIC Lfcin (17-30) (50 µl); Group II, 10^7^ CFU of MDR- EAEC (50 µl) with 2X MIC Lfcin (17-30) (50 µl); Group III, 10^7^ CFU of MDR- EAEC (50 µl) with Meropenem (10 µg/ml; 50 µl); Group IV, 10^7^ CFU of MDR- EAEC (50 µl) with CA-MH broth (50 µl). Similar groups were also made for the other two MDR-EAEC isolates. The respective groups along with the appropriate controls were incubated at 37^0^C up to 72 h.

**S7. Melanisation assay**

To determine the melanin production, which is an immune marker, the *G. mellonella* (n=3 larvae per group) were investigated at an interval of 6 h pi for 24 h, followed by 24 h interval till 96 h. An aliquot of the pooled haemolymph (100 µl) was transferred into 96-well microtiter plate for measuring the optical density (OD) at a wavelength of 450 nm using ELISA plate reader (Thermo Scientific Multiskan GO) with OD values of haemolymph collected from apparently healthy un-inoculated larvae as the background control.

**S8. Enumeration of MDR-EAEC counts**

The haemolymph of *G. mellonella* (n=3 larvae per group) was collected aseptically at an interval of 6 h pi up to 24 h, followed by 24 h interval till 96 h pi in sterile NSS. The haemolymph was vortexed thoroughly and serially diluted 10-fold times in sterile NSS and the bacterial burden was assessed on EMB agar plates supplemented with 100 µg of ampicillin per plate [4]. The number of MDR-EAEC colonies on the plates was counted and expressed as log_10_CFU/ml of haemolymph.

**S9. Enumeration of haemocytes**

The haemocyte density of *G. mellonella* (n= 3 larvae per group) at an interval of 6 h pi up to 24 h, followed by 24 h interval till 96 h pi were quantified. No attempt was made to discriminate between the different haemocyte subtypes.

**S10.LDH cytotoxicity assay**

*G. mellonella* (n=3 larvae per group) were analysed for the production of LDH, as a marker of cell damage, at an interval of 6 h pi for 24 h, followed by 24 h interval till 96 h pi using QuantiChrom LDH cytotoxicity assay kit, according to the manufacturer’s instructions. The positive and negative controls used in this study were distilled water and 20% Triton X-100, respectively. The absorbance was read using ELISA plate reader at 500 nm; the haemolymph from untreated larvae served as background control and the cytotoxicity was calculated as, Cytotoxicity (%) = (OD_Sample_ - OD_Control_)/ (OD_Total Lysis_ - OD_Control_) x 100, wherein, sample is the control absorbance of treated cell; control is the experimental absorbance of the untreated cell control and total lysis is the absorbance of Triton X-100 treated cells.

**S11. Histopathology**

To study the tissue-level changes, the larvae at each time point were subjected to histopathological examination observing standard laboratory protocols. The neutral buffered formalin (10%)-fixed whole larvae were dissected transversally into two halves utilizing anatomic pincers and by using a new lancet blade for each larva. The procedure was carefully performed to avoid the squeeze of the larval tissues; each paraffin-embedded sections (3 µm) was then sectioned and stained using Haematoxylin and Eosin (H&E) to evaluate tissue morphology. The microscopic visualization was performed (Leica Microscope DMLB) and the image acquisition was carried out (NanoZoomer-XR C12000, Hamamatsu Photonics, Japan).

As the infection progressed at 24 h p.i., a comparatively higher cluster of haemocytes was observed in the sub-cuticular area of group I; fat bodies with points of melanisation and increased load of bacteria were also seen around the tubular organelle (Supplementary Fig. 4). In groups II and III, mild accumulation of bacteria was observed around the organelle with a distribution of scanty haemocytes exhibiting no noticeable aggregates or melanisation. In contrast, groups IV and V looked healthy with individually distributed scanty haemocytes exhibiting no noticeable aggregates or melanisation. Further, at 48 h p.i., the clustered haemocytes in the sub-cuticular area and fat bodies with evidence of melanisation and an increased bacterial load around tubular organelle were observed in group I. However, in group II, aggregates of haemocytes with points of melanisation were noticed in the sub-cuticular area with a very mild accumulation of bacteria around the organelle (Supplementary Fig.4); while, in group III, scanty distribution of haemocytes with no noticeable aggregates or melanisation were observed with a mild accumulation of bacterial load around the organelle. Surprisingly, other larval groups (groups IV and V) exhibited individually distributed scanty haemocytes with no noticeable aggregates or melanisation. Later, at 72 h p.i., a mild accumulation of bacteria was appreciated in group I with a comparative reduction in the melanisation and haemocyte aggregates within the fat bodies. Moreover, in group II, melanisation as well as the aggregates of haemocytes were reduced with a mild accumulation of bacteria (Supplementary Fig.4); while scanty distribution of haemocytes with no noticeable aggregates or melanisation as well as reduced haemocyte accumulation was noticed in group III. Interestingly, all the other groups (treatment and control) exhibited scanty haemocytes with no noticeable aggregates or melanisation.

**S12. Biofilm forming ability of MDR-EAEC strains**

**S12.1 Congo Red Binding Assay**

The biofilm-producing ability of MDR-EAEC isolates was assessed using Congo Red binding assay [8]. The inoculated Congo red agar (CRA) plates showing black streaks or colonies with a dry crystalline consistency were regarded as strong to moderate biofilm producers, whereas the presence of pink or red colonies was regarded to be weak biofilm producers.

**S12.2 Microbial adhesion to Solvents (MATS)**

The hydrophobicity index was determined by microbial adhesion to solvents [9] using three solvents (n-hexadecane, chloroform, and diethyl ether). The suspension of MDR-EAEC cells grown overnight in nutrient broth (OD_600_ nm of 0.80) was prepared in 1.5 mM sodium chloride (A_0_); 2.4 ml of this suspension was overlaid with 0.4 ml of each solvent. After vigorous mixing, phases were allowed to separate for 15 min at room temperature and the OD_600_ nm of aqueous phase (A_1_) was measured. The percentage of hydrophobicity was calculated as, hydrophobicity (%) = [1- (A_1/_ A_0_)] x 100.

All the three MDR-EAEC strains displayed a maximal affinity for the acidic solvent (chloroform) and a low affinity for the basic solvent (diethyl ether) despite having similar van der Waal’s properties (Supplementary Fig. 6). With apolar solvent (n-hexadecane), a moderate affinity (ca. 40%) was observed.

**S12.3 Time-dependent biofilm-forming ability in different media**

Time-dependent biofilm-forming ability of the test isolates in different media was assessed using a microtiter plate assay [10,11]. DMEM and nutrient broth each supplemented with either 0.45% D–Glucose or 0.45% D–Mannose were used. *E. coli* ATCC 25922 and *E. coli* DH5α were used as positive and negative biofilm controls, respectively. The optical density values obtained from the test cultures with each respective medium and time were statistically analyzed to determine the optimal medium and the time required for biofilm formation and also to classify the test cultures as high, moderate, and weak biofilm producers [11].

**Table S1**: Physico-chemical properties of Lfcin (17-30)

| **Sl No.** | **Peptide designation** | **Amino acid sequence** | **Length** | **Class** | **Molecular weight** | **Extinction coefficient** | **Hydrophobic residue (%)** | **Net Charge (at pH 7.0)** | **Isoelectric point** | **Mechanism of action** |
| --- | --- | --- | --- | --- | --- | --- | --- | --- | --- | --- |
| 1. | Lfcin (17-30) | FKCRRWQWRMKKLG | 14 | Beta Sheet | 1922.04 | 11125 | 42.0 | 5.95 | 12.26 | Inner membrane depolarization;  Permeability of cytoplasm |

**Table S2**: *In vitro* antibiotic susceptibility testing of MDR-EAEC isolates.

| **NCBI GenBank Accession No.** | **Antibiotic Susceptibility testing** | | | | | | | | | | **MIC Values (mg/L)** | | | | | | | | |
| --- | --- | --- | --- | --- | --- | --- | --- | --- | --- | --- | --- | --- | --- | --- | --- | --- | --- | --- | --- |
|  | **CIP** | **CTR** | **AMP** | **COT** | **TE** | **IMI** | **SXT** | **GEN** | **CL** | **CO** | **CIP** | **CTR** | **AMP** | **COT** | **TE** | **IMI** | **GEN** | **CL** | **CO** |
| **KY941936.1 (MDR 1)** | S | S | R | R | R | S | R | S | S | S | S | S | >240 | >240 | 60 | S | S | S | S |
| **KY941937.1**  **(MDR 2)** | R | R | R | R | R | S | S | S | S | S | 120 | >240 | >240 | >240 | 2 | S | S | S | S |
| **KY941938.1**  **(MDR 3)** | R | R | R | R | R | S | R | S | S | S | 60 | >240 | >240 | >240 | 30 | S | S | S | S |

CIP- Ciprofloxacin, CTR- Ceftriaxone, AMP- Ampicillin, COT- Co-trimoxazole, TE- Tetracycline, IMI- Imipenem, SXT- Sulfamethoxazole, GEN- Gentamicin, CL- Chloramphenicol, CO- Colistin sulphate, S – Sensitive, R- Resistant.

**Table S3A**. *In vitro* thermostability observed for Lfcin (17-30) against MDR-EAEC isolates

| **Temperature**  **90^0^C** | **Incubation Time**  **(min)** | **Lfcin (17-30)** | | |
| --- | --- | --- | --- | --- |
|  |  | **MDR 1** | **MDR 2** | **MDR 3** |
| **MIC (mg/L)** | **5** | 32.0 | 32.0 | 32.0 |
|  | **15** | 32.0 | 32.0 | 32.0 |
|  | **30** | 32.0 | 32.0 | 32.0 |
| **MBC (mg/L)** | **5** | 32.0 | 32.0 | 32.0 |
|  | **15** | 32.0 | 32.0 | 32.0 |
|  | **30** | 32.0 | 32.0 | 32.0 |

| **Temperature**  **70^0^C** | **Incubation Time**  **(min)** | **Lfcin (17-30)** | | |
| --- | --- | --- | --- | --- |
|  |  | **MDR 1** | **MDR 2** | **MDR 3** |
| **MIC (mg/L)** | **5** | 32.0 | 32.0 | 32.0 |
|  | **15** | 32.0 | 32.0 | 32.0 |
|  | **30** | 32.0 | 32.0 | 32.0 |
| **MBC (mg/L)** | **5** | 32.0 | 32.0 | 32.0 |
|  | **15** | 32.0 | 32.0 | 32.0 |
|  | **30** | 32.0 | 32.0 | 32.0 |

**Table S3B**. *In vitro* protease (trypsin) stability observed for Lfcin (17-30) against MDR-EAEC isolates.

| **TRYPSIN** | **Incubation Time**  **(min)** | **Lfcin (17-30)** | | |
| --- | --- | --- | --- | --- |
|  |  | **MDR 1** | **MDR 2** | **MDR 3** |
| **MIC (mg/L)** | **0.5** | 16.0 | 16.0 | 16.0 |
|  | **2** | 16.0 | 16.0 | 16.0 |
|  | **5** | 16.0 | 16.0 | 16.0 |
|  | **15** | 16.0 | 16.0 | 16.0 |
|  | **30** | 16.0 | 16.0 | 16.0 |
| **MBC (mg/L)** | **0.5** | 16.0 | 16.0 | 16.0 |
|  | **2** | 16.0 | 16.0 | 16.0 |
|  | **5** | 16.0 | 16.0 | 16.0 |
|  | **15** | 16.0 | 16.0 | 32.0 |
|  | **30** | 16.0 | 16.0 | 32.0 |

**Table S3B**. *In vitro* protease (Proteinase-K) stability observed for Lfcin (17-30) against MDR-EAEC isolates.

| **PROTEINASE-K** | **Incubation Time**  **(min)** | **Lfcin (17-30)** | | |
| --- | --- | --- | --- | --- |
|  |  | **MDR 1** | **MDR 2** | **MDR 3** |
| **MIC (mg/L)** | **0.5** | 8.0 | 8.0 | 4.0 |
|  | **2** | 8.0 | 8.0 | 4.0 |
|  | **5** | 8.0 | 8.0 | 4.0 |
|  | **15** | 8.0 | 8.0 | 4.0 |
|  | **30** | 8.0 | 8.0 | 2.0 |
| **MBC (mg/L)** | **0.5** | 16.0 | 32.0 | 4.0 |
|  | **2** | 16.0 | 32.0 | 4.0 |
|  | **5** | 16.0 | 32.0 | 4.0 |
|  | **15** | 16.0 | 32.0 | 4.0 |
|  | **30** | 8.0 | 16.0 | 4.0 |

**Table S3C**. *In vitro* salt stability observed for Lfcin (17-30) against MDR-EAEC isolates.

| **NaCl**  **(150 mM)** | **Lfcin (17-30)** | | |
| --- | --- | --- | --- |
|  | **MDR 1** | **MDR 2** | **MDR 3** |
| **MIC (mg/L)** | 32.0 | 32.0 | 32.0 |
| **MBC (mg/L)** | 32.0 | 32.0 | 32.0 |

| **MgCl_2_**  **(2 mM)** | **Lfcin (17-30)** | | |
| --- | --- | --- | --- |
|  | **MDR 1** | **MDR 2** | **MDR 3** |
| **MIC (mg/L)** | 32.0 | 32.0 | 32.0 |
| **MBC (mg/L)** | 32.0 | 32.0 | 32.0 |

**Table S4**. *In vitro* cytotoxicity of Lfcin (17-30) at different MIC concentrations against sheep RBCs.

|  | **Haemolysis (%)** |
| --- | --- |
| **MIC (1X)** | 0.0 |
| **MIC (2X)** | 0.0 |
| **MIC (4X)** | 1.71 |

**A**

**B**

**Fig. S1**. *In vitro* cytotoxicity observed for Lfcin (17-30) at different concentrations on (A) HEp-2 and (B) RAW 264.7 cells.

**Fig. S2**. ***In vitro* effect of Lfcin (17-30) on commensal gut flora**. Untreated *L. acidophilus* and *L. rhamnosus* serve as positive growth control while media (MRS broth) serve as a negative control.

**
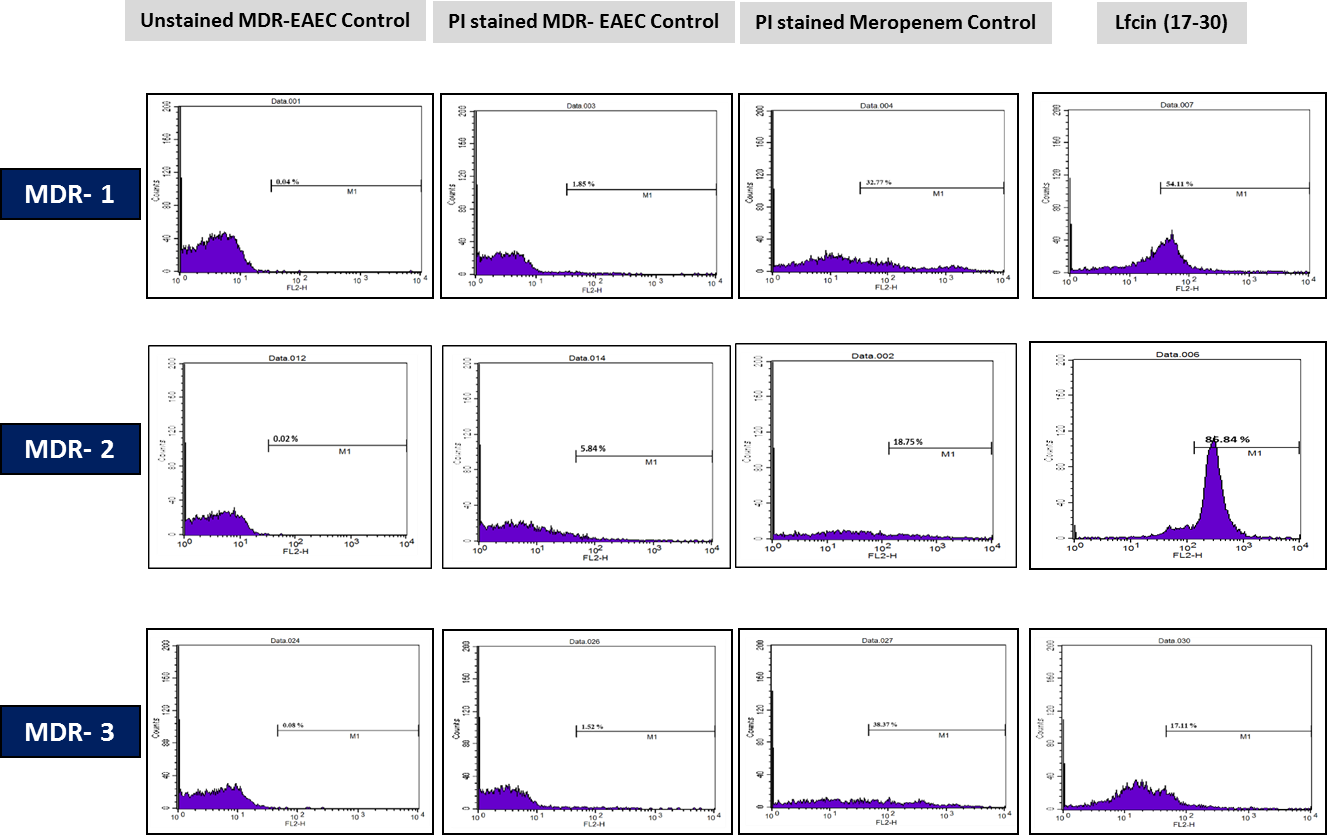
**

**Fig. S3**. ***In vitro* membrane permeability of Lfcin (17-30) against MDR-EAEC strains by flow cytometry**. Histograms reveal propidium iodide (PI) uptake by MDR-EAEC on exposure to MIC levels of Lfcin (17-30) with appropriate control groups; rows represent three MDR-EAEC strains tested, while columns denote the bacterial cells exposed to meropenem and AMP.


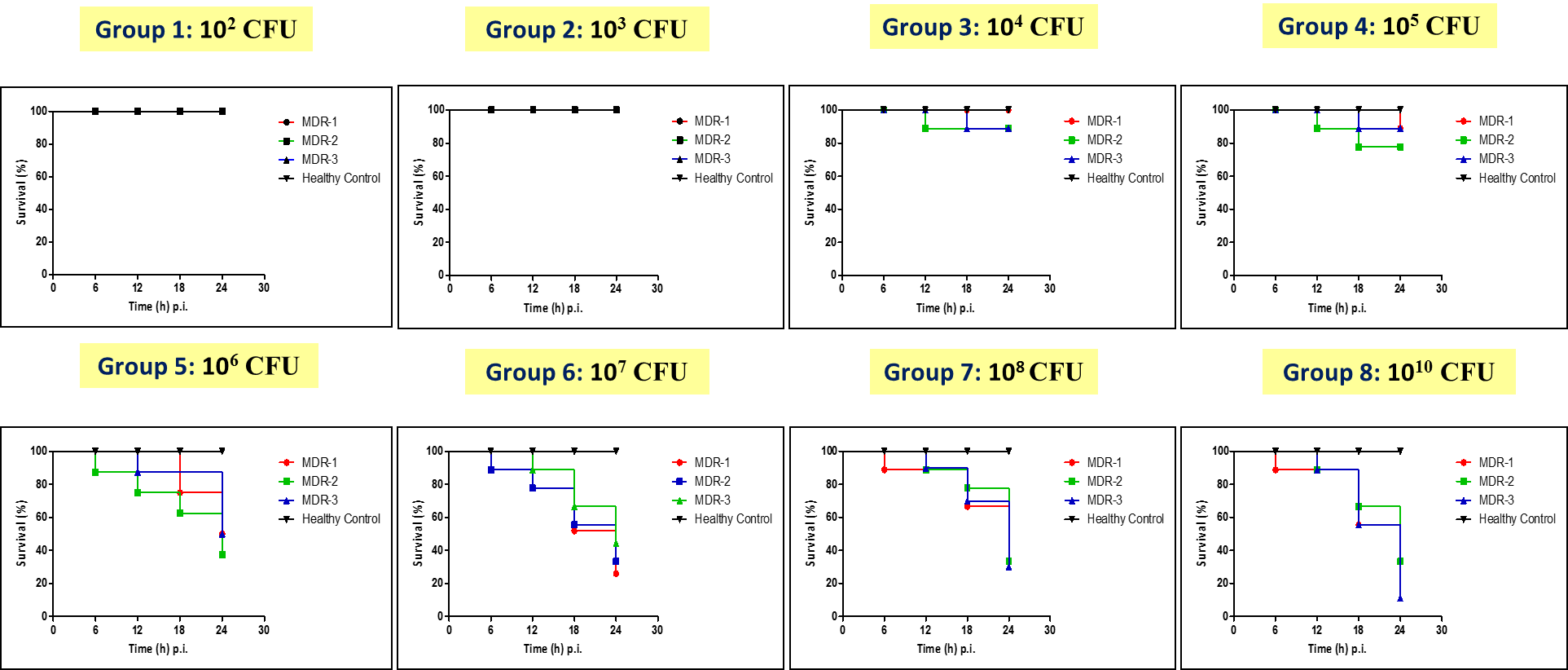


**Fig. S4**. **MDR-EAEC infection of *Galleria mellonella* larvae**. Larvae were infected with 3 different MDR-EAEC strains and injected with 8 different doses of bacteria. All results represent means of at least 3 independent experiments with 9 larvae per treatment. Survival curves were plotted using Kaplan-Meier method and statistical analysis was performed using the log-rank test for multiple comparisons (GraphPad Software, San Diego, CA).


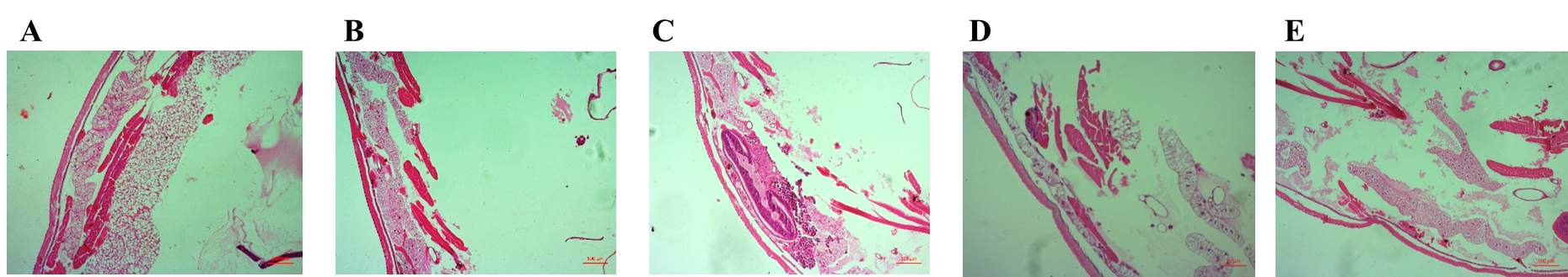


**Fig. S5**. **Histopathological analysis of *G. mellonella* larvae exhibiting the effect of Lfcin (17-30) on MDR-EAEC *in vivo* using H&E staining at 24 h pi**. The images denote larvae inoculated with PBS (A), uninfected larvae treated with Lfcin (17-30) (B), LD_50_ dose of MDR-EAEC (C), MDR-EAEC-induced larvae treated with Meropenem 3 h pi (D), MDR-EAEC-induced larvae treated with Lfcin (17-30) 3 h p.i. (E). Representative images are shown from histological analysis of 3 larvae for each condition from 3 independent experiments. Scale bars, 100 µm.


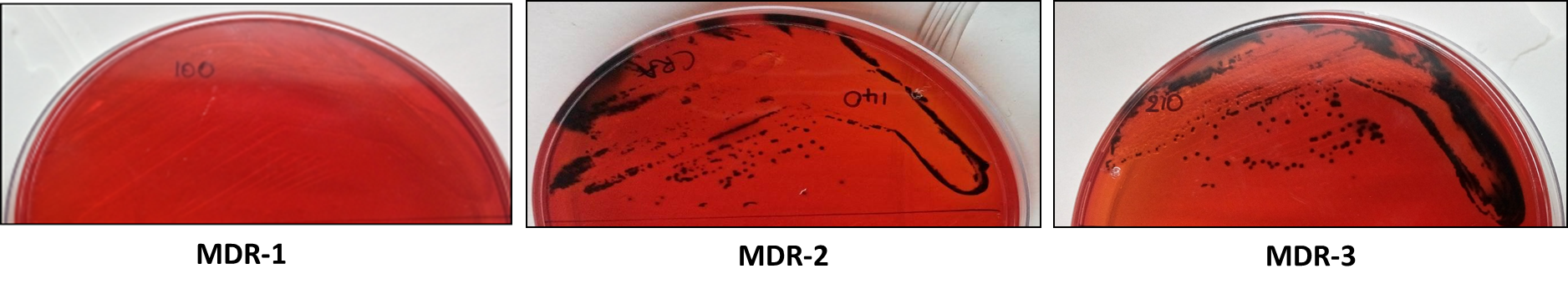


**Fig. S6. Congo red binding assay of MDR-EAEC isolates**

**Fig. S7. Adhesion of MDR-EAEC to various solvent-water interfaces.**

**A**

**B**

**C**

**Fig. S8. Comparative biofilm-forming ability of MDR-EAEC strains on different media (A), surfaces (B), and time intervals (C) using Crystal Violet microtiter plate assay.** DMEM HG: DMEM (0.45% D-Glucose); DMEM HM: DMEM (0.45% D-Mannose); NB: Nutrient Broth; NB HG: Nutrient Broth (0.45% D-Glucose); NB HM: Nutrient Broth (0.45% D- Mannose).

**
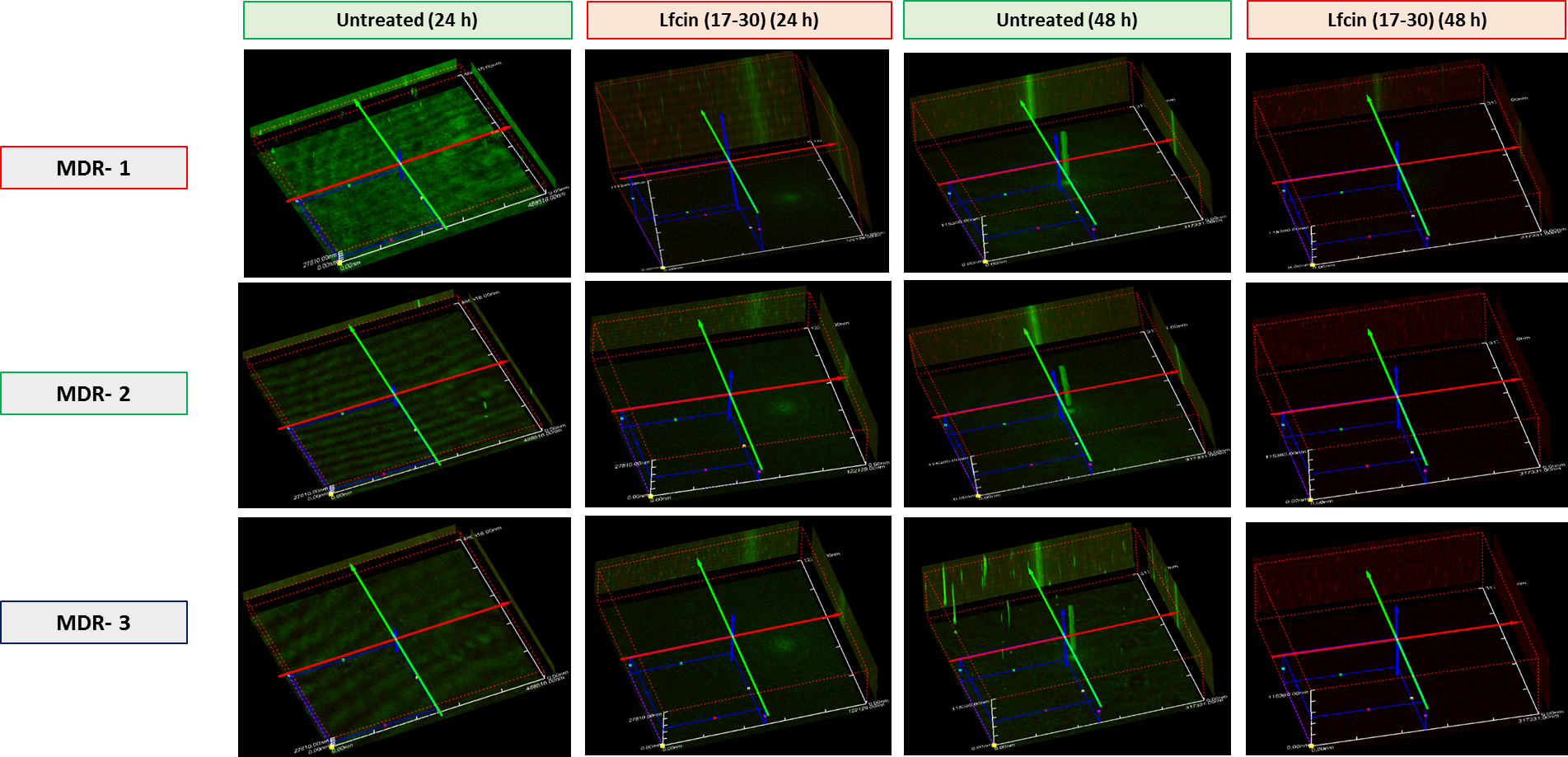
**

**Fig. S9. Inhibition of MDR-EAEC biofilm by Lfcin (17-30) at 24 h and 48 h.** Biofilms of MDR-EAEC were processed using Live/Dead staining, observed by the red and green filters of the Confocal microscope, and merged. Each row indicates MDR-1, MDR-2, and MDR-3 EAEC strains; the first column of each time interval indicates untreated MDR-EAEC strains while the second column indicates MDR-EAEC strains treated with Lfcin (17-30).

**
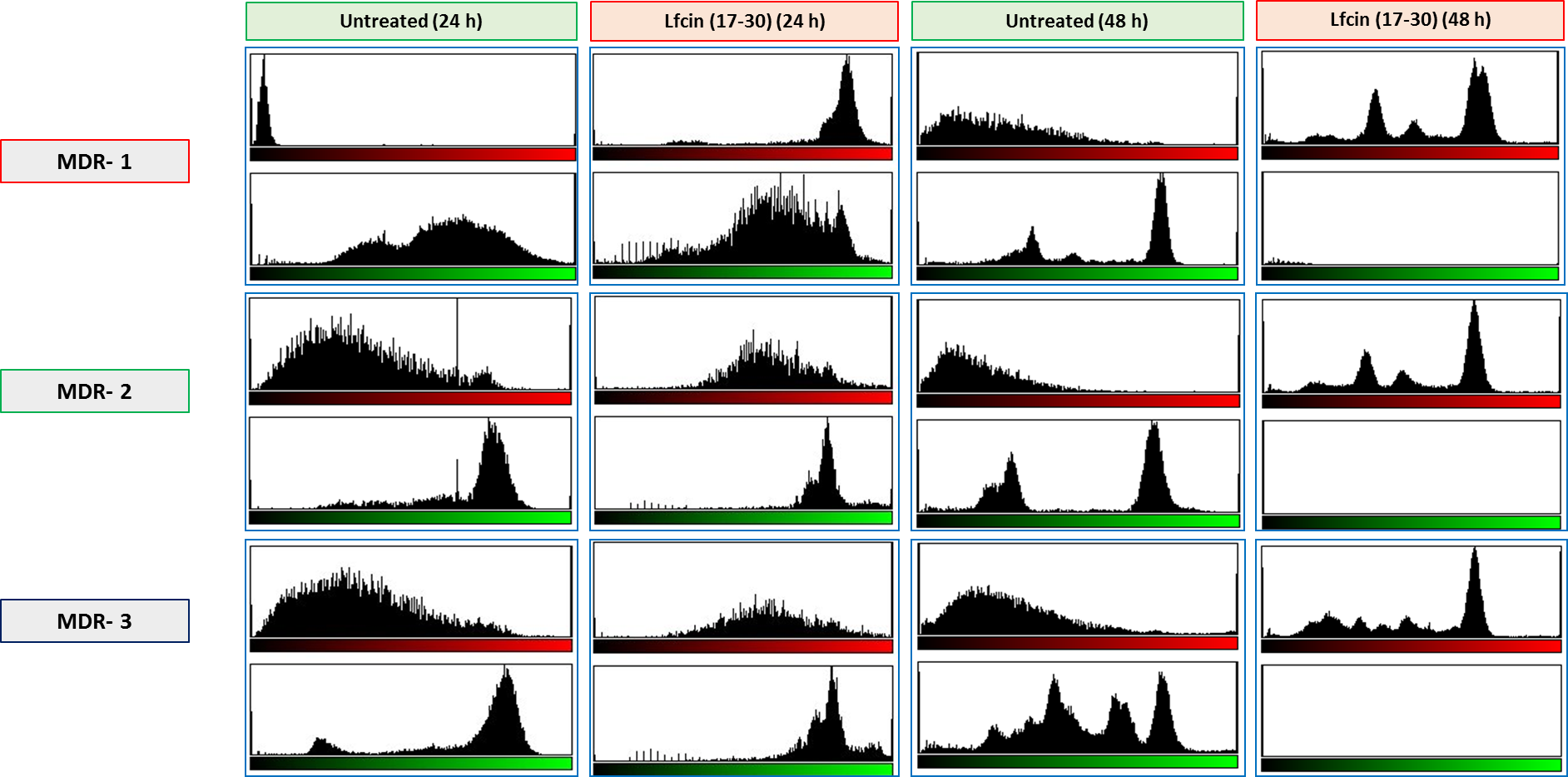
**

**Fig. S9. Inhibition of MDR-EAEC biofilm by Lfcin (17-30) at 24 h and 48 h.** Live/Dead intensity histogram of confocal images using Fiji ImageJ software ver. 1.51s; the first column of each time interval indicates untreated MDR-EAEC strains while the second column indicates MDR-EAEC strains treated with Lfcin (17-30). The peak underscored red indicates dead bacterial intensity and green indicates live intensity.

**
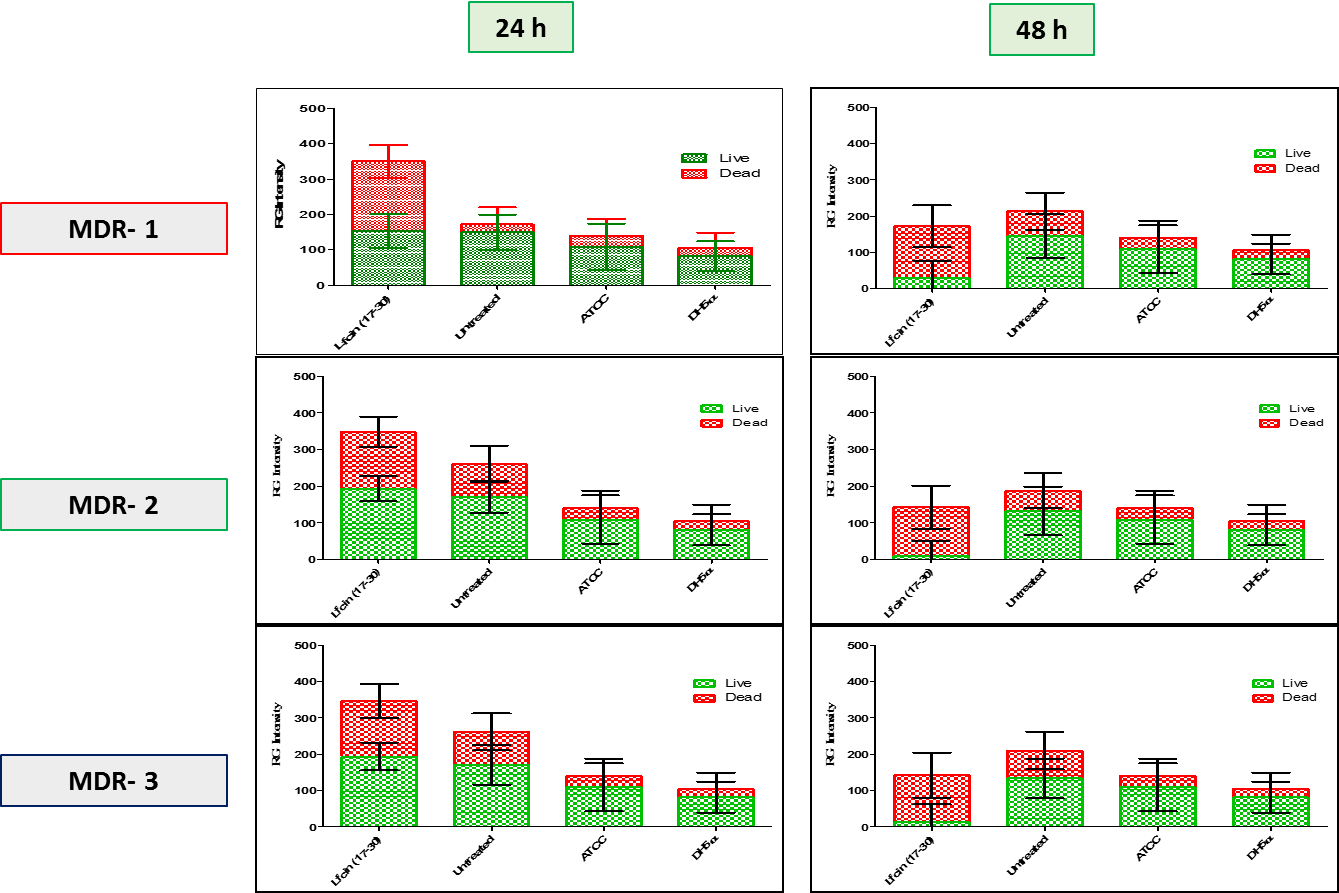
**

**Fig. S9. Red/Green intensity plot of MDR-EAEC biofilm inhibition by Lfcin (17-30) at 24 h and 48 h.** MDR-EAEC biofilm inhibition [(A) MDR-1, (B) MDR-2, and (C) MDR-3] at 24 h and 48 h; error bars indicate the standard deviation between strains. Positive and negative control bars indicate corresponding untreated MDR-EAEC and DH5α biofilms, respectively without AMP.

**
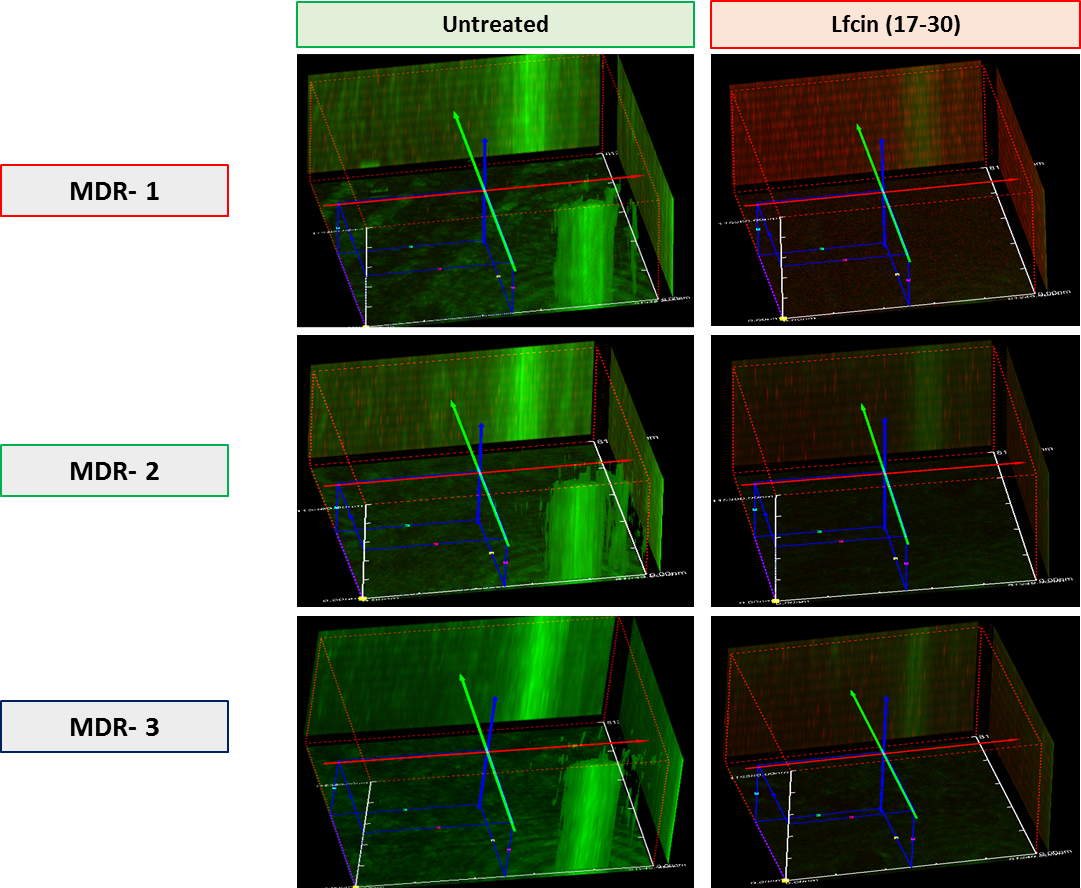
**

**Fig. S10. Effect of treating MDR-EAEC preformed biofilms (48 h) with Lfcin (17-30) for an additional 24 h.** Biofilms of MDR-EAEC were processed using Live/Dead staining, observed by the red and green filters of the Confocal microscope, and merged. Each row indicates MDR-1, MDR-2, and MDR-3 EAEC strains; the first column of each time interval indicates untreated MDR-EAEC strains while the second column indicates MDR-EAEC strains treated with Lfcin (17-30).

**
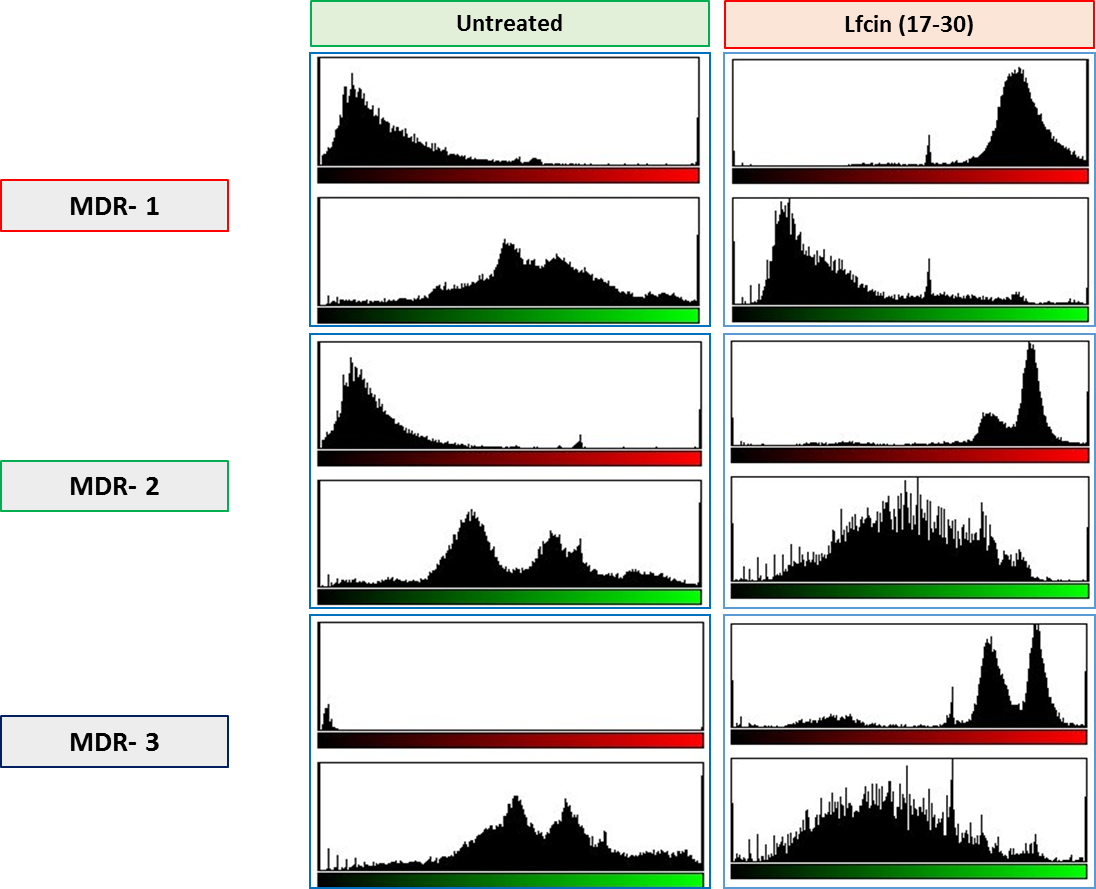
**

**Fig. S10. Effect of treating MDR-EAEC preformed biofilms (48 h) with Lfcin (17-30) for an additional 24 h.** Live/Dead intensity histogram of confocal images using Fiji ImageJ software ver. 1.51s; the first column of each time interval indicates untreated MDR-EAEC strains while the second column indicates MDR-EAEC strains treated with Lfcin (17-30). The peak underscored red indicates dead bacterial intensity and green indicates live intensity.

**
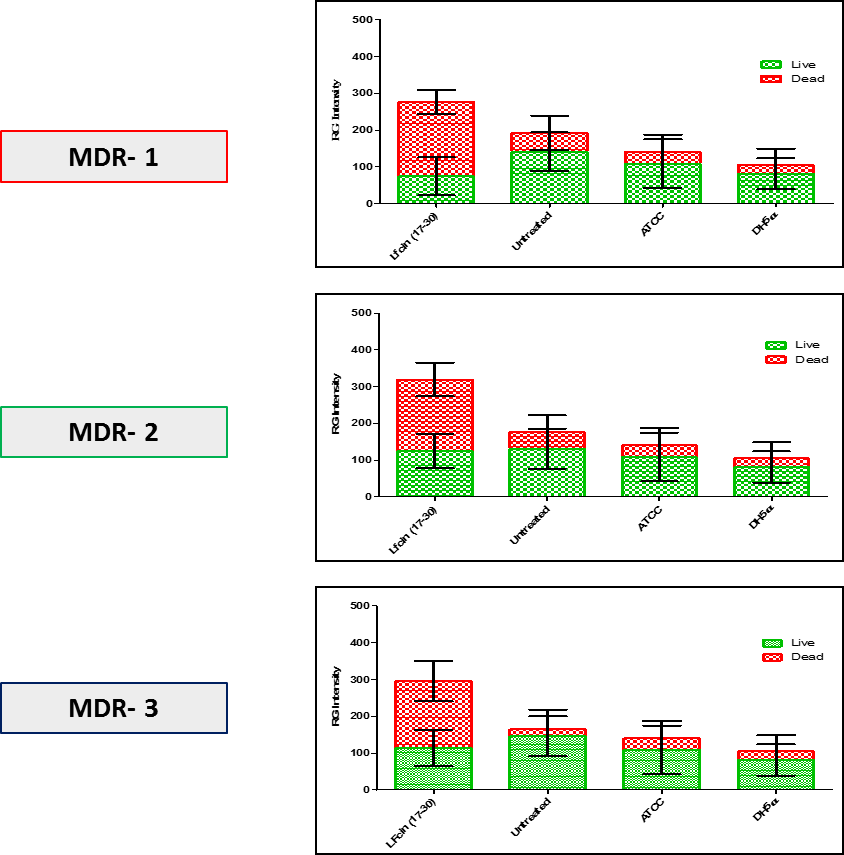
**

**Fig. S10. Red/Green intensity plot of MDR-EAEC preformed biofilms (48 h) treated with Lfcin (17-30) for an additional 24 h.** Eradication of MDR-EAEC [(A) MDR-1, (B) MDR-2, and (C) MDR-3] preformed biofilm; error bars indicate the standard deviation between strains. Positive and negative control bars indicate corresponding untreated MDR-EAEC and DH5α biofilms, respectively without AMP.

**References**:

1. Clinical and Laboratory Standards Institute [CLSI] (2018). Performance Standards for Antimicrobial Susceptibility Testing, 28th Edn, Wayne, PE: CLSI. <https://clsi.org/media/2663/m100ed29_sample.pdf>.
2. Ebbensgaard, A., Mordhorst, H., Overgaard, M. T., Nielsen, C. G., Aarestrup, F. M., and Hansen, E.B. 2015. Comparative evaluation of the antimicrobial activity of different antimicrobial peptides against a range of pathogenic bacteria. *PLoS One*. 10, e0144611. [doi: 10.1371/journal.pone.0144611](https://doi.org/10.1371/journal.pone.0144611)
3. Vaucher, R. A., De da Motta, S. A., and Brandelli, A. (2010). Evaluation of the *in vitro* cytotoxicity of the antimicrobial peptide P34. *Cell Biol. Int.* 34, 317-323. doi: 10.1042/CBI20090025
4. Miles, A. A., Misra, S. S., and Irwin, J. O. (1938) The estimation of the bactericidal power of the blood. *Epidemiol. Infect.* 38(6), 732-749.
5. Zhang SK, Song JW, Gong F, Li SB, Chang HY, Xie HM et al. Design of an α-helical antimicrobial peptide with improved cell-selective and potent anti-biofilm activity. Sci Rep 2016; 6:27394.
6. Marri L, Dallai R, Marchini D. The novel antibacterial peptide ceratotoxin A alters permeability of the inner and outer membrane of *Escherichia coli* K-12. Curr Microbiol 1996; 33:40-43.
7. Epand RF, Pollard JE, Wright JO, Savage PB, Epand RM. Depolarization, bacterial membrane composition, and the antimicrobial action of ceragenins. Antimicrob Agents Chemother 2010; 54:3708-13.
8. Freeman, D. J., Falkiner, F. R., and Keane, C. T. (1989) New method for detecting slime production by coagulase-negative staphylococci. *J. Clin. Path*. 42(8), 872-874. DOI: [doi.org/10.1136/jcp.42.8.872](http://dx.doi.org/10.1136/jcp.42.8.872).
9. Bellon-Fontaine, M. N., Rault, J., and Van Oss, C. J. (1996) Microbial adhesion to solvents: a novel method to determine the electron-donor/electron-acceptor or Lewis acid-base properties of microbial cells. *Colloids and Surfaces B: Biointerfaces,* 7(1-2), 47-53. DOI: [doi.org/10.1016/0927-7765 (96)01272-6](https://doi.org/10.1016/0927-7765(96)01272-6).
10. Wakimoto, N., Nishi, J., Sheikh, J., Nataro, J. P., Sarantuya, J. A. V., Iwashita, M., and Kawano, Y. (2004) Quantitative biofilm assay using a microtiter plate to screen for enteroaggregative *Escherichia coli*.  *Am. J. Trop. Med. Hyg*. 71(5), 687-690. DOI: [doi.org/10.4269/ajtmh.2004.71.687](https://doi.org/10.4269/ajtmh.2004.71.687).
11. Vijay, D., Dhaka, P., Vergis, J., Negi, M., Mohan, V., Kumar, M., Malik, S.V.S., Barbuddhe, S. B., and Rawool, D.B., (2015) Characterization and biofilm forming ability of diarrhoeagenic enteroaggregative *Escherichia coli* isolates recovered from human infants and young animals. *Comp. Immunol. Microbiol. Infect. Dis.* 38, 21-31. DOI: [doi.org/10.1016/j.cimid.2014.11.004](https://doi.org/10.1016/j.cimid.2014.11.004).
